# Block-pulse integrodifference equations

**DOI:** 10.1007/s00285-023-01986-6

**Published:** 2023-09-13

**Authors:** Nora M. Gilbertson, Mark Kot

**Affiliations:** Department of Applied Mathematics, University of Washington, Seattle, WA USA

**Keywords:** Integrodifference equations, Block-pulse series, Allee effects, Population dynamics, Spatial ecology, 37N25, 92D25, 92D40

## Abstract

We present a hybrid method for calculating the equilibrium population-distributions of integrodifference equations (IDEs) with strictly increasing growth, for populations that are confined to a finite habitat-patch. This method is based on approximating the growth function of the IDE with a piecewise-constant function, and we call the resulting model a block-pulse IDE. We explicitly write out analytic expressions for the iterates and equilibria of the block-pulse IDEs as sums of cumulative distribution functions. We characterize the dynamics of one-, two-, and three-step block-pulse IDEs, including formal stability analyses, and we explore the bifurcation structure of these models. These simple models display rich dynamics, with numerous fold bifurcations. We then use three-, five-, and ten-step block-pulse IDEs, with a numerical root finder, to approximate models with compensatory Beverton–Holt growth and depensatory, or Allee-effect, growth. Our method provides a good approximation for the equilibrium distributions for compensatory and depensatory growth and offers numerical and analytical advantages over the original growth models.

## Introduction

In recent decades, integrodifference equations (IDEs) have become a popular tool for analyzing the spatial dynamics of biological populations. They have been used to explore many problems in spatial ecology, including estimating speeds of invasion (e.g., Kot et al. 1996; Wang et al. 2002; Gagnon et al. 2015), population persistence on a finite habitat patch (e.g., Kot and Schaffer 1986; Van Kirk and Lewis 1997; Reimer et al. 2016), and critical speeds of climate-change-driven range shifts (e.g., Zhou and Kot 2011; Harsch et al. 2014; Cobbold and Stana 2020).

IDEs are spatially nonlocal models (Lee et al. 2001) that are discrete in time and continuous in space. They are especially useful for modeling the dynamics of species that have non-overlapping generations and distinct growth and dispersal stages (Kot and Schaffer 1986; Lutscher 2019). The basic IDE for a population with discrete and nonoverlapping generations is

1 $$\begin{aligned} n_{t+1}\left( x\right) = \int _{\Omega } k\left( x, y\right) g\left[ n_t\left( y\right) \right] dy, \end{aligned}$$

where $n_t\left( x\right)$ is the population density in generation *t* at a spatial location *x*, $\Omega$ is the spatial domain, $k\left( x, y\right)$ is the dispersal kernel, and $g\left[ n_t\left( y\right) \right]$ is the growth or recruitment function. Population density is mapped from the current generation to the next in two distinct phases. First, the population grows, while remaining fixed in space. In particular, $g\left[ n_t\left( y\right) \right]$ gives the new population-density at location *y* after growth has occurred. After growth, individuals disperse, relocating from starting location *y* to new location *x* with probability governed by $k\left( x, y\right)$. The dispersal kernel is a probability density function for the final location *x* of the individuals.

In contrast to reaction–diffusion models, which are continuous in both time and space, IDE models account for seasonal growth and can incorporate a variety of dispersal patterns, including long-distance dispersal (Lewis et al. 2016). The ability to choose a dispersal kernel that best fits the population being modeled is a key feature of IDEs. As a result, IDE models have the advantage of increased ecological realism, as many species possess the features of seasonal growth and long-distance dispersal (Okubo and Levin 1989; Andersen 1991; Kot et al. 1996; Lutscher 2019).

Despite the advantages of IDEs, these models have drawbacks as well. IDEs can be computationally expensive since, as for most spatially nonlocal models, individuals may disperse long distances (Lutscher 2019). At the extreme end of this spatial complexity, under certain conditions, a propagule may start from any location in the spatial domain of the model and disperse to any other location in that domain.

In addition, it is often difficult to write down exact solutions for the equilibria or to perform general analytical explorations, except in certain special cases (Kot and Schaffer 1986; Zhou and Kot 2011; Bramburger and Lutscher 2019; Lutscher 2019). This analytic difficulty arises because finding equilibria amounts to finding functions that are fixed points of nonlinear integral equations. Finally, most analyses of IDEs lack information about unstable equilibria, as unstable equilibria do not show up in standard numerical experiments. While an argument may be made that it is more relevant to focus on (asymptotically) stable equilibria, in the interest of a complete characterization of the model it would be useful to observe all equilibria.

In order to circumvent some of these difficulties in analyzing IDE models, a number of tools have been developed to approximate or simplify the original model. Special cases, such as an IDE model with a separable kernel or with the Laplace kernel, may allow for analytical results (Kot and Schaffer 1986; Zhou and Kot 2011; Bramburger and Lutscher 2019; Lutscher 2019). Other tools for analyzing IDE models involve various approximations of the dispersal kernel, which can be used to approximate steady states and eigenvalues of IDEs, and may be employed for models with one or more species. These methods include average dispersal-success (Van Kirk and Lewis 1997, 1999; Fagan and Lutscher 2006), modified average dispersal-success (Reimer et al. 2016), and geometric symmetrization (Kot and Phillips 2015; Phillips and Kot 2015; Rinnan 2018a, b; Marcinko 2020).

In this paper, we focus on an alternative for analyzing the population dynamics of a single species confined to a finite spatial domain. In contrast to the methods discussed above, which approximate the dispersal kernel, we approximate the growth function. Drawing inspiration from early work by Mark Lewis on the infinite-domain problem (Kot et al. 1996) and more recent developments by Otto (2017) and Nestor and Li (2022), we use a piecewise-constant function to approximate the growth function. We refer to the IDE with this approximated growth as a block-pulse IDE. An *m*-step block-pulse IDE has *m* terms, or steps, in the block-pulse approximation. This terminology does not preclude using block-pulse approximations for the dispersal kernel as well; indeed, many applications of block-pulse methods for solving other integral problems involve approximating the entire integrand rather than a single component (Jiang and Schaufelberger 1992; Babolian et al. 2008).

The piecewise-constant nature of the growth function makes the block-pulse IDE simple and analytically tractable. This formulation of the IDE removes many of the analytical barriers mentioned earlier and also offers numerical advantages. With the increased tractability of the block-pulse IDE, we are able to obtain analytic expressions for both the iterates and the equilibria of the model. Of particular interest, we gain explicit formulas for not only the stable but also the unstable equilibria of the block-pulse model.

The analytic formulas for the block-pulse equilibria involve a set of constants whose values are found by using a numerical root-finder to solve a finite, nonlinear set of implicit equations. In other words, we use block-pulse IDEs as part of a hybrid analytical–numerical method for analyzing IDEs. The numerical component, however, is small, and involves solving for a relatively small number of values. Our method is thus more numerically efficient than a pure root-finding method for an operator equation, while the dominant analytic part of the method provides significant insight regarding model equilibria.

Furthermore, this method can be used for a broad set of growth functions, including growth functions with Allee effects. An Allee effect, or depensation, occurs when a population’s per-capita recruitment increases, with density, at low densities (Allee et al. 1949; Lewis and Kareiva 1993). Integrodifference equations with Allee effects have, historically, been challenging to analyze (Lutscher 2019). However, the block-pulse method allows us to analyze both simple forms of growth, like compensatory growth, as well as challenging forms of growth, such as depensatory growth.

In Sect. 2, we look at block-pulse series and show how they can be used to approximate growth functions. In Sect. 3, we describe the block-pulse IDE model and discuss assumptions and properties of the model, including a general, closed-form, analytic expression for the iterates of the spatial population-distributions. In Sect. 4, we perform formal analyses of one-, two-, and three-step block-pulse IDEs and determine general patterns for these models. Our analyses include equilibrium solutions, stability and bifurcation analyses, and explorations of parameter space. In Sect. 5, we generalize these analytic results to the *m*-step case and we describe the hybrid analytical–numerical method for block-pulse IDEs. In Sect. 6, we explore an *m*-step block-pulse IDE subject first to compensatory Beverton–Holt dynamics and then to growth with a depensatory Allee effect. Sections 5 and 6 are the most applied sections and the applied reader may find it useful to look at these sections before returning to Sect. 4. In Sect. 7, we describe the implications of our results and discuss future research.

This work provides an analytically tractable method for analyzing population dynamics on a finite domain. For a large number of steps in the block-pulse series, we find that the block-pulse IDE model offers a good approximation to the original model. Furthermore, the block-pulse IDE model also brings significant analytical advantages and an improvement in numerical efficiency for a reasonable number of steps. The framework outlined here provides a simple, effective means for exploring population dynamics in a finite-domain IDE, and offers a novel way to investigate the impact of Allee effects.

## Block-pulse series

### Block-pulse functions, series, and properties

Block-pulse functions are an orthogonal set of disjoint functions that have piecewise-constant values. These functions have traditionally been used for a variety of problems in engineering, in particular in systems science and control (Rao 1983; Jiang and Schaufelberger 1992). Block-pulse functions have been extensively applied to developing numerical and analytical methods for solving a number of integral problems (e.g., Babolian et al. 2008; Maleknejad et al. 2011; Ebadian and Khajehnasiri 2014; Balcı and Sezer 2016). Block-pulse functions have simple operations and a number of useful properties that make a series of these functions highly useful in studying problems with integrals or derivatives (Jiang and Schaufelberger 1992).

A set of block-pulse functions $\phi _i\left( n\right) , i = 1, 2,\ldots , m$ is conventionally defined on the interval $n \in \left[ 0, N\right)$ as

2 $$\begin{aligned} \phi _i\left( n\right) = {\left\{ \begin{array}{ll} 1, &{} \left( i - 1\right) h \le n < ih \\ 0, &{} \text {otherwise}, \end{array}\right. } \end{aligned}$$

where $h = N/m$ and *m* is some positive number corresponding to the total number of steps in the block-pulse series. Block-pulse functions are disjoint and orthogonal with each other, and they form a complete basis for real, bounded, square-integrable functions on the interval $\left[ 0, N\right)$ as $m \rightarrow \infty$ (Rao 1983; Jiang and Schaufelberger 1992).

Based on these properties, we can expand any real, bounded, continuous function $g\left( n\right)$ that is square integrable over the domain $n \in \left[ 0, N\right)$ into a block-pulse series. The functions $g\left( n\right)$ that we consider describe population growth, and so we choose the upper domain-limit *N* to be a population limit governed by the carrying capacity of the population. An *m*-step block-pulse series for the function $g\left( n\right)$ is

3 $$\begin{aligned} g\left( n\right) \approx \sum _{i = 1}^{m} g_i \, \phi _i\left( n\right) , \quad i = 1, 2,\ldots , m, \end{aligned}$$

where $g_i$ is the block-pulse coefficient for the *i*th block-pulse function $\phi _i\left( n\right)$. Each coefficient $g_i$ is simply the average value of the function $g\left( n\right)$ over the *i*th subinterval, so that

4 $$\begin{aligned} g_i = \frac{1}{h} \int _{\left( i - 1\right) h}^{ih} g\left( n\right) dn. \end{aligned}$$

As each function $\phi _i\left( n\right)$ is disjoint from the others, the series in Eq. (3) is thus a piecewise-constant approximation to the original function $g\left( n\right)$, with the value of the constants governed by the original function. The block-pulse series will converge pointwise to the original function as the number of steps $m \rightarrow \infty$ (Jiang and Schaufelberger 1992).

As we will be expanding growth functions into block-pulse series, we note that there may be compelling ecological reasons to deviate from the general formula for the block-pulse coefficients given by Eq. (4). In particular, a reasonable growth function $g\left( n\right)$ should have ${g\left( 0\right) = 0}$, so that a population does not grow from nothing. However, even if the original function satisfies this requirement, Eq. (4) does not guarantee that the corresponding block-pulse series will as well. To be consistent with the conventional definition of a block-pulse series, we will primarily use Eq. (4) to calculate our block-pulse coefficients. An alternative that deviates from the block-pulse convention but that allows for population extinction would be to force ${g_1 = 0}$. We briefly explore this possibility in Sect. 6.2.

### Block-pulse approximations to a growth function

To illustrate the block-pulse approximation method, consider the nonspatial model ${n_{t+1} = g\left( n_t\right) }$ with a strictly-increasing growth-function,

5 $$\begin{aligned} g\left( n_t\right) = \frac{\left[ \left( 1 + \rho ^2\right) /K\right] n_t^2}{1 + \left( \rho /K\right) ^2 n_t^2}, \end{aligned}$$

with an Allee effect, defined on the interval ${n_t \in \left[ 0, N\right) }$ with growth parameter $\rho$ and carrying capacity *K*, where ${N = K = 1}$. In the nonspatial model with this growth function, there are three equilibria $n_{t+1} = n_t = n$: two stable fixed-points at ${n = 0}$ and ${n = K}$ and one unstable fixed-point, the Allee threshold, at ${n = K/\rho ^2}$.

To compare the original growth-function with its block-pulse series, we choose five steps for our approximation. The five-step block-pulse series is given by

6 $$\begin{aligned} g\left( n_t\right) \approx {\left\{ \begin{array}{ll} g_1, &{} 0 \le n_t< N/5 \\ g_2, &{} N/5 \le n_t< 2N/5 \\ g_3, &{} 2N/5 \le n_t< 3N/5 \\ g_4, &{} 3N/5 \le n_t< 4N/5 \\ g_5, &{} 4N/5 \le n_t < N, \\ \end{array}\right. } \end{aligned}$$

with $g_{i} < g_{i+1}$ for $i = 1, 2, 3, 4$. Each coefficient $g_i$ is the density of recruits or the growth level when the population density $n_t$ is within the given limits. The population densities *iN*/5 are density thresholds. Passing through each threshold leads to a change in the level of recruitment.

**Fig. 1:**
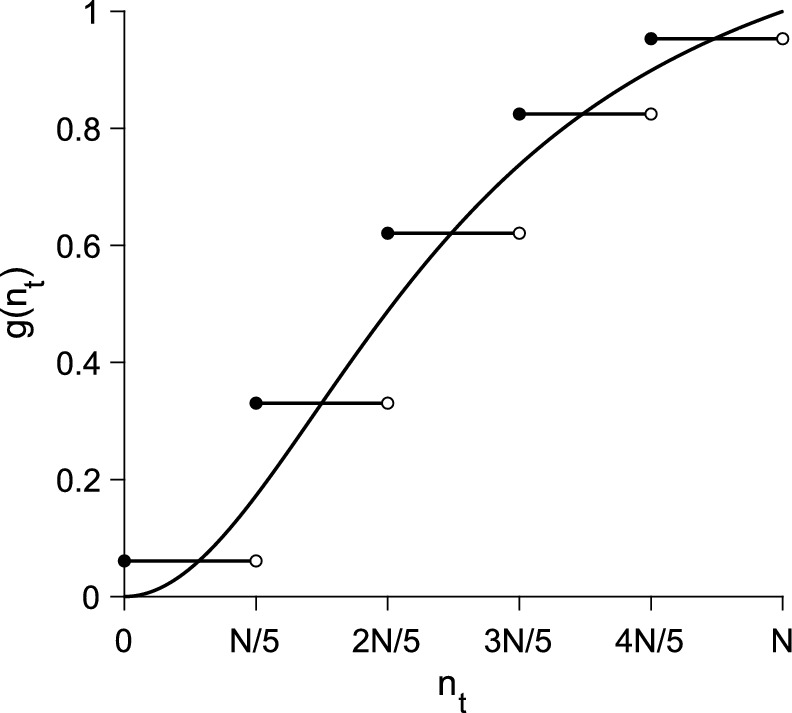
The Allee growth-function from Eq. (5) with the five-step block-pulse approximation from Eq. (6). Parameters are ${\rho = 2}$, ${K = 1}$, ${N =1}$, and the five growth-levels are ${g_1 \approx 0.06}$, ${g_2 \approx 0.33}$, ${g_3 \approx 0.62}$, ${g_4 \approx 0.82}$, ${g_5 \approx 0.95}$

Figure 1 shows the Allee growth-function along with its five-step block-pulse approximation. At each of the density thresholds, we see that the growth function jumps to the next level of recruitment. With this illustration of the block-pulse method in place, we now turn to the block-pulse IDE itself.

## Block-pulse IDE

We begin by examining the model from Eq. (1), where the population is confined to a finite, stationary, one-dimensional patch of habitat of length *L* such that ${\Omega = \left[ -L/2, \ L/2\right] }$. We assume that habitat outside the patch is hostile and that individuals that disperse outside of the patch do not reproduce. For mathematical simplicity, as we examine the usefulness of the block-pulse method, we assume that the growth function $g\left[ n_t\left( y\right) \right]$ is strictly increasing. We also assume that dispersal is homogeneous and isotropic. Thus, the dispersal kernel is symmetric and can be rewritten as a difference kernel, $k\left( x, y\right) = k\left( x-y\right)$. This difference kernel may be interpreted as a probability density function for the displacement of individuals in the population.

Under these assumptions, the model from Eq. (1) can be written as

7 $$\begin{aligned} n_{t+1}\left( x\right) = \int _{-L/2}^{L/2} k\left( x-y\right) g\left[ n_t\left( y\right) \right] dy. \end{aligned}$$

The population density at location *x* in the next generation $t+1$, $n_{t+1}\left( x\right)$, is thus given by the convolution of a growth function with a dispersal kernel. We primarily use the Laplace distribution for our dispersal kernel as it accounts for some long-distance dispersal and possesses a number of useful properties. At the same time, our approach is not limited to this distribution (see Sect. 6.2).

We assume that our initial population-distribution is symmetric and unimodal. Though we recognize that such an assumption may not be true in general in nature, for the time being we make this assumption for mathematical simplicity. Applying an increasing growth-function to a symmetric and unimodal distribution will result in a symmetric and unimodal population-distribution after growth. We also assume that the dispersal kernel is unimodal. Thus, we are taking the convolution of two symmetric, unimodal distributions, which will also be symmetric and unimodal (Wintner 1938; Dharmadhikari and Joag-Dev 1988; Purkayastha 1998). Many dispersal kernels satisfy the conditions of being symmetric and unimodal, including the popular Gaussian, Laplace, and Cauchy kernels. Using these assumptions, the population distributions given by the model will always be symmetric and unimodal, with the peak (population) density occurring at the center of the patch at $x = 0$ and the minimum density occurring at the edges of the patch, at $x = \pm L/2$.

We have constructed the simplest possible case for the population distributions of our IDE model using the above assumptions. This baseline case now allows us to explore the practicality of the block-pulse IDE and to identify and generalize any analytic patterns that emerge in the dynamics of our block-pulse models.

With these assumptions in hand, we consider an *m*-step block-pulse IDE. This IDE, with its growth function given by a block-pulse series, may be written as

8 $$\begin{aligned} n_{t+1}\left( x\right)&= \int _{-L/2}^{L/2} k\left( x-y\right) \sum _{i=1}^{m} g_i \, \phi _i\left[ n_t\left( y\right) \right] dy \nonumber \\&= \sum _{i=1}^{m} g_i \int _{-L/2}^{L/2} k\left( x-y\right) \phi _i\left[ n_t\left( y\right) \right] dy. \end{aligned}$$

As mentioned in Sect. 2.2, the coefficients $g_i$ are different growth levels. As we have assumed our growth function to be strictly increasing, we have $g_i < g_{i+1}$ for $i = 1, 2,\ldots , m-1$.

Recall that each block-pulse function $\phi _i$ is nonzero only over a limited spatial region where it is equal to one. We may expand Eq. (8) into a sum of at most ${2m - 1}$ integrals, where each integral is defined only over the spatial region(s) where ${\phi _i \ne 0}$ for each *i*. The boundaries of each region, ${x = \pm r_{i, \, t}}$, are the spatial thresholds where the population threshold is reached, ${n_t\left( \pm r_{i, \, t}\right) = ih}$. After expanding Eq. (8), using a change of variables ${u = x - y}$, and rewriting the resulting integrals in terms of cumulative distribution functions, we obtain the simple expression

9 $$\begin{aligned} n_{t+1}\left( x\right)&= g_1\left[ F\left( x + L/2\right) - F\left( x - L/2\right) \right] \nonumber \\&\quad + \left( g_2 - g_1\right) \left[ F\left( x + r_{1, \, t}\right) - F\left( x - r_{1, \, t}\right) \right] + \cdots \nonumber \\&\quad + \left( g_m - g_{m-1}\right) \left[ F\left( x + r_{m-1, \, t}\right) - F\left( x - r_{m-1, \, t}\right) \right] , \end{aligned}$$

or

10 $$\begin{aligned} n_{t+1}\left( x\right)&= g_1\left[ F\left( x + L/2\right) - F\left( x - L/2\right) \right] \nonumber \\&\quad + \sum _{i = 1}^{m-1} \left( g_{i+1} - g_i\right) \left[ F\left( x + r_{i, \, t}\right) - F\left( x - r_{i, \, t}\right) \right] , \end{aligned}$$

where $F\left( x\right)$ is the cumulative distribution function (CDF) of a probability density function $k\left( z\right)$, defined as

11 $$\begin{aligned} F\left( x\right) = \int _{-\infty }^{x} k\left( z\right) dz. \end{aligned}$$

We note that reducing our IDE model to Eqs. (9) or (10) amounts to approximating a Riemann–Stieltjes integral with a Riemann–Stieltjes sum. Due to Arzelà’s (or Osgood’s) bounded convergence theorem (Hildebrandt 1963; Luxemburg 1971; Apostol 1974; Monteiro et al. 2016, 2019), this series has nice convergence properties as $m \rightarrow \infty$.

The symmetry of the spatial thresholds $x = \pm r_{i, \, t}$ occurs because we have assumed our population distributions to be symmetric and unimodal. Thus, each spatial threshold where the population passes through a density threshold will occur twice in a symmetric fashion, if it occurs at all. We have $r_{i, \, t} > r_{i+1, \, t}$, with the *t* subscript denoting the time dependence of the spatial thresholds.

For this general example, we have assumed that the population passes through all density thresholds inside of the patch $\left[ -L/2, L/2\right]$ and sees all *m* growth levels. Note that the population may fail to pass through a density threshold or may pass through it at a spatial threshold outside of the patch, in which case Eq. (10) has fewer terms.

We now have a model that is expressed entirely in terms of cumulative distribution functions, so that we have explicit analytic expressions for the entire spatial population-distribution in the next generation. This makes the block-pulse IDE analytically and numerically approachable, and the CDF form of the IDE is immensely useful. We now turn to a formal analysis of one-, two-, and three-step block-pulse IDEs.

## Analytical results

As seen above, the block-pulse IDE may be rewritten into a remarkably simple form. Given the assumptions laid out in Sect. 3, block-pulse IDEs are a class of models with much more analytical tractability than most IDE models. We will now explore explicit equilibrium solutions and their regions of validity in parameter space, stability of equilibria, and bifurcations in one-, two-, and three-step block-pulse IDEs. In order to facilitate analysis, we will fix some of the block-pulse coefficients $g_i$ to take on specific values. In later sections, where we apply the block-pulse IDE to particular growth functions, these growth levels are all explicitly given by Eq. (4) and the parameters of the original growth function.

Throughout the rest of this paper, we will illustrate examples using the Laplace kernel and its CDF, though the general trends we observe hold for other dispersal kernels satisfying our assumptions as well (see Sect. 6.2). The probability density function for the Laplace distribution is

12 $$\begin{aligned} k\left( z\right) = \frac{1}{2}\alpha e^{-\alpha |z |}, \end{aligned}$$

and the cumulative distribution function is

13 $$\begin{aligned} F\left( z\right) = {\left\{ \begin{array}{ll} \frac{1}{2} e^{\alpha z}, &{} z < 0 \\ 1 - \frac{1}{2} e^{-\alpha z}, &{} z \ge 0, \end{array}\right. } \end{aligned}$$

where $1/\alpha$ is the average dispersal-distance of the population.

### One-step block-pulse IDE

The one-step block-pulse IDE has a growth function approximated by the single constant

14 $$\begin{aligned} g\left( n_t\right) \approx g_1, \quad 0 \le n_t < N. \end{aligned}$$

The corresponding block-pulse IDE is

15 $$\begin{aligned} n_{t+1}\left( x\right)&= g_1 \int _{-L/2}^{L/2} k\left( x - y \right) dy \nonumber \\&= g_1 \left[ F\left( x + L/2\right) - F\left( x - L/2\right) \right] , \end{aligned}$$

where the second equality is obtained after using the same change of variables, $u = x - y$, and method of rewriting the integral in terms of cumulative distribution functions as in Sect. 3.

It is clear, from Eq. (15), that the population reaches its carrying capacity in one generation and stays there for all time *t*, regardless of the initial density of the population. We thus have a stable equilibrium at

16 $$\begin{aligned} n\left( x\right) = g_1 \left[ F\left( x + L/2\right) - F\left( x - L/2\right) \right] . \end{aligned}$$

**Fig. 2:**
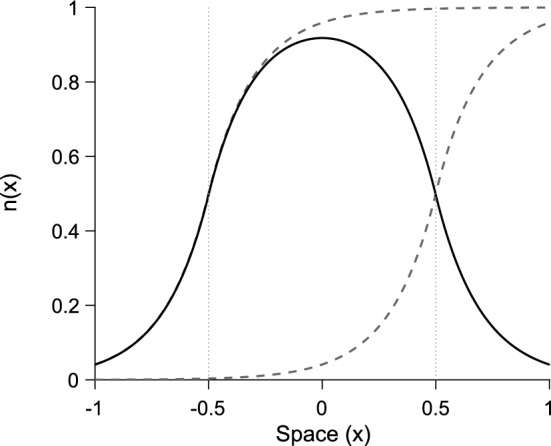
The equilibrium distribution from Eq. (16) (solid) along with the two component cumulative distribution functions (dashed). The left dashed distribution is the first term ${F\left( x + L/2\right) }$, the right dashed distribution is the second term ${F\left( x - L/2\right) }$. Vertical dotted lines indicate the spatial domain ${x \in \left[ -L/2, L/2\right] }$. Parameters are ${L = 1}$, ${N = 1}$, ${g_1 = 1}$, ${\alpha = 5}$

The shape of the equilibrium distribution is simply the difference between two shifted cumulative distribution functions, as shown in Fig. 2.

This equilibrium is valid for ${n\left( x\right) < N}$. Since the maximum population-density occurs at $x = 0$, we thus require ${n\left( 0\right) < N}$ for existence of the equilibrium. Substituting $x = 0$ into Eq. (16) and rearranging, we thus need

17 $$\begin{aligned} g_1 < \frac{N}{F\left( L/2\right) - F\left( -L/2\right) }. \end{aligned}$$

### Two-step block-pulse IDE

**Fig. 3:**
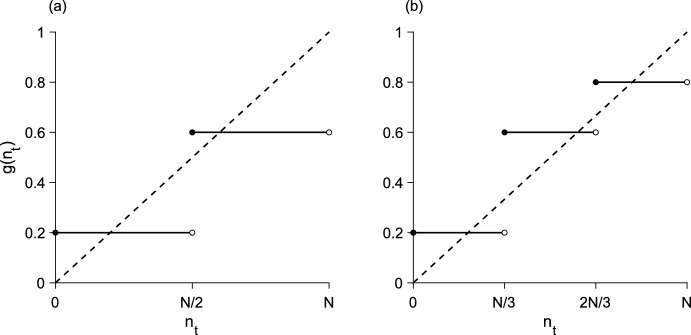
Block-pulse growth-functions with **a** two steps and **b** three steps. The growth functions (solid) are overlaid on the line of equality (dashed). Parameter values are ${N = 1}$, ${g_1 = 0.2}$, ${g_2 = 0.6}$, ${g_3 = 0.8}$

The growth-function approximation for the two-step block-pulse IDE is

18 $$\begin{aligned} g\left( n_t\right) \approx {\left\{ \begin{array}{ll} g_1, &{} 0 \le n_t< N/2 \\ g_2, &{} N/2 \le n_t < N, \end{array}\right. } \end{aligned}$$

with $g_1 < g_2$. The population density $n_t = N/2$ is a density threshold. Below the threshold, the population grows to $g_1$, and above it, recruitment jumps to $g_2$. This growth function is illustrated in Fig. 3a.

We expect three equilibrium solutions of the block-pulse IDE—one with a low growth-level everywhere in the patch, one with a high growth-level everywhere in the patch, and one with both growth-levels in the patch. We suspect initially that the two one-growth-level equilibria, those with a single growth-level in the patch, should be stable.

While the analysis for the one-step model was trivial, the two-step model contains more interesting dynamics. Depending on the location and existence of the spatial-threshold points ${x = \pm r_{1, \, t}}$, where ${n\left( \pm r_{1, \, t}\right) = N/2}$, the equilibrium solution to the two-step block-pulse IDE may take on one of three different forms, matching our initial intuition. These three cases are addressed separately in the following sections.

#### Low equilibrium

In the first case, the population never attains the density threshold of *N*/2. The population grows at the low level $g_1$ everywhere and the spatial thresholds do not exist. The corresponding IDE can be written as

19 $$\begin{aligned} n_{t+1}\left( x\right)&= g_1 \int _{x - L/2}^{x + L/2} k\left( z \right) dz \nonumber \\&= g_1 \left[ F\left( x + L/2\right) - F\left( x - L/2\right) \right] . \end{aligned}$$

It is clear that the population distribution in Eq. (19) is always the same regardless of *t*. Thus, there is a stable equilibrium, which we call the low equilibrium, at

20 $$\begin{aligned} n\left( x\right) = g_1 \left[ F\left( x + L/2\right) - F\left( x - L/2\right) \right] . \end{aligned}$$

This is a valid equilibrium so long as ${n\left( 0\right) < N/2}$, at which point we would transition into a bridge equilibrium (Sect. 4.2.3). Substituting $x = 0$ into Eq. (20) and rearranging to solve for the growth level gives a condition of

21 $$\begin{aligned} g_1 < \frac{N/2}{F\left( L/2\right) - F\left( -L/2\right) } = g_a. \end{aligned}$$

**Fig. 4:**
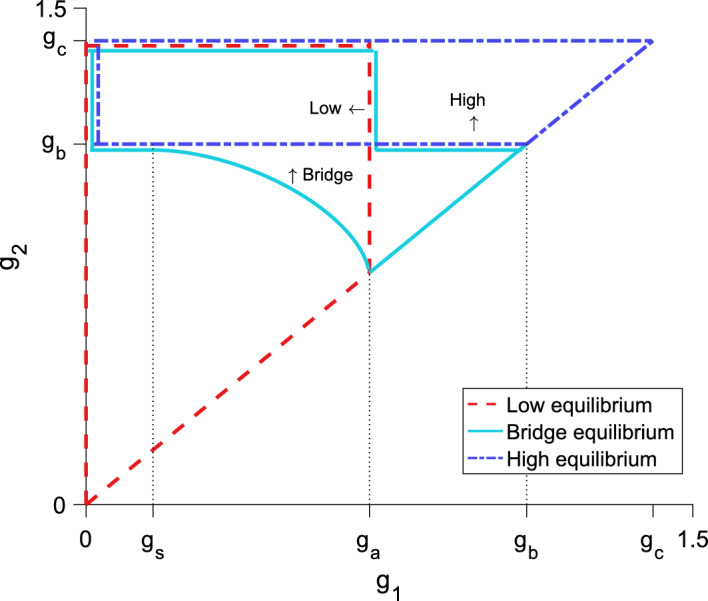
Regions of validity in parameter space for the three equilibrium forms. All equilibria are invalid for ${g_1 \ge g_2}$. The low equilibrium is valid inside the dashed region, the high equilibrium is valid inside the dashed-dotted region, and the bridge equilibrium is valid inside the solid region. Vertical dotted lines indicate the boundaries in $g_1$ that lead to different behaviors of the bridge equilibrium. For ${g_{s}< g_1 < g_a}$, as $g_2$ increases through the lower boundary for the bridge-equilibrium region, a fold bifurcation occurs, resulting in two bridge equilibria for $g_2$ values above the boundary and below $g_b$. At $g_b$, the upper bridge-equilibrium becomes a high equilibrium. Parameter values are ${L = 0.5}$, ${N = 1}$, ${\alpha = 5}$, ${g_s \approx 0.17}$, ${g_a \approx 0.7}$, ${g_b \approx 1.1}$, ${g_c \approx 1.4}$

Fig. 4 illustrates the region in parameter space where this equilibrium is valid.

#### High equilibrium

In the second case, the population is above *N*/2 everywhere in the patch and grows at high level $g_2$ inside the patch. The spatial thresholds exist outside the patch, so that

22 $$\begin{aligned} -r_{1, \, t}< -L/2< L/2 < r_{1, \, t}. \end{aligned}$$

The IDE in this case is

23 $$\begin{aligned} n_{t+1}\left( x\right)&= g_2 \int _{x - L/2}^{x + L/2} k\left( z \right) dz \nonumber \\&= g_2 \left[ F\left( x + L/2\right) - F\left( x - L/2\right) \right] . \end{aligned}$$

The only difference between this mapping and the mapping in Sect. 4.2.1 is replacing $g_1$ with $g_2$.

The population distribution does not change regardless of *t*. There is a stable equilibrium, called the high equilibrium, given by

24 $$\begin{aligned} n\left( x\right) = g_2 \left[ F\left( x + L/2\right) - F\left( x - L/2\right) \right] . \end{aligned}$$

In order for this to be a valid equilibrium, it must be above *N*/2 everywhere in the patch, or ${n\left( L/2\right) > N/2}$. Substituting ${x = L/2}$ into Eq. (24) and simplifying, we require

25 $$\begin{aligned} g_2 > \frac{N/2}{F\left( L\right) - F\left( 0\right) } = g_b. \end{aligned}$$

We also require ${n\left( 0\right) < N}$, or

26 $$\begin{aligned} g_2 < \frac{N}{F\left( L/2\right) - F\left( -L/2\right) } = g_c. \end{aligned}$$

Thus for ${g_b< g_2 < g_c}$ there is a stable high-equilibrium. See Fig. 4 for an illustration of the region of validity.

#### Bridge equilibrium

The third case occurs when the population distribution inside the patch is both above and below *N*/2, so that the population sees both growth levels inside the patch. The spatial thresholds are inside the patch, with

27 $$\begin{aligned} -L/2< -r_{1, \, t}< r_{1, \, t} < L/2. \end{aligned}$$

The IDE now takes the form

28 $$\begin{aligned} n_{t+1}\left( x\right) = g_1 \int _{x + r_{1, \, t}}^{x + L/2} k\left( z \right) dz + g_2 \int _{x - r_{1, \, t}}^{x + r_{1, \, t}} k\left( z \right) dz + g_1 \int _{x - L/2}^{x - r_{1, \, t}} k\left( z \right) dz, \end{aligned}$$

which simplifies to the mapping

29 $$\begin{aligned} n_{t+1}\left( x\right)&= g_1 \left[ F\left( x + L/2\right) - F\left( x -L/2 \right) \right] \nonumber \\&\quad + \left( g_2 - g_1\right) \left[ F\left( x + r_{1, \, t}\right) - F\left( x - r_{1, \, t}\right) \right] . \end{aligned}$$

**Fig. 5:**
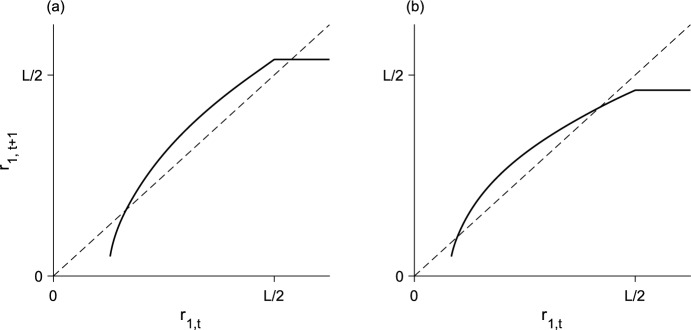
Spatial-threshold mapping from Eq. (30) (solid curve for ${r_{1, \, t} < L/2}$) for two different parameter sets with **a** one fixed point and **b** two fixed points. The solid horizontal line for ${r_{1, \, t} > L/2}$ is an equivalent ${r_{1, \, t} - r_{1, \, t+1}}$ mapping for the high map; as the high map does not depend on ${r_{1, \, t}}$ the corresponding ${r_{1, \, t+1}}$ is constant regardless of ${r_{1, \, t}}$. Using asymptotic Lyapunov stability of the spatial-threshold fixed-points as a proxy for Lyapunov stability of the full equilibrium distributions, in **a**, the high equilibrium is stable and the bridge equilibrium is unstable; in **b** the lower bridge-equilibrium is unstable and the upper bridge-equilibrium is stable. Given a particular $r_{1, \, t}$ in the implicit map from Eq. (30), $r_{1, \, t+1}$ was computed with the bisection method. Parameter values are ${L = 0.5}$, ${N = 1}$, ${\alpha = 5}$, **a** ${g_1 = 0.4}$, ${g_2 = 1.2}$; **b** ${g_1 = 0.6}$, ${g_2 = 1}$

Given $n_{t+1}\left( x\right)$, we can compute the next spatial threshold $r_{1, \, t+1}$. That is, we have an implicit mapping from $r_{1, \, t}$ to $r_{1, \, t+1}$ given by

30 $$\begin{aligned} n_{t+1}\left( r_{1, \, t+1}\right)&= g_1 \left[ F\left( r_{1, \, t+1} + L/2\right) - F\left( r_{1, \, t+1} -L/2 \right) \right] \nonumber \\&\quad + \left( g_2 - g_1\right) \left[ F\left( r_{1, \, t+1} + r_{1, \, t}\right) - F\left( r_{1, \, t+1} - r_{1, \, t}\right) \right] = N/2, \end{aligned}$$

where we have used the fact that $n_{t+1}\left( r_{1, \, t+1}\right) = N/2$. This map is illustrated in Fig. 5.

The fixed points of this map occur when ${r_{1, \, t+1} = r_{1, \, t} = r_1}$, so that

31 $$\begin{aligned} g_1 \left[ F\left( r_{1} + L/2\right) - F\left( r_{1} -L/2 \right) \right] + \left( g_2 - g_1\right) \left[ F\left( 2r_{1}\right) - F\left( 0\right) \right] = N/2. \end{aligned}$$

These fixed points are evident in Fig. 5 as the intersections of the $r_{1, \, t}$ mapping with the line of equality. When the spatial threshold is at a fixed point $r_1$, the entire population-distribution is at equilibrium so that ${n_{t+1}\left( x\right) = n_t\left( x\right) = n\left( x\right) }$. Substituting the fixed point $r_1$ into Eq. (29), we therefore have an equilibrium population-distribution, which we call the bridge equilibrium, at

32 $$\begin{aligned} n\left( x\right) = g_1 \left[ F\left( x + L/2\right) - F\left( x -L/2 \right) \right] + \left( g_2 - g_1\right) \left[ F\left( x + r_{1}\right) - F\left( x - r_{1}\right) \right] . \end{aligned}$$

The number of spatial-threshold fixed-points and their stability corresponds to the number and stability of the bridge equilibria.

When discussing stability of the equilibria, we are referring specifically to asymptotic Lyapunov stability. In the current context, however, our notion of stability is somewhat restrictive, as we apply our stability analyses only to unimodal and symmetric distributions and initial conditions.

The bridge equilibrium will be valid for ${n\left( L/2\right)< N/2< n\left( 0\right) < N}$. To facilitate analysis of the bridge equilibrium, we solve for $g_2$ in Eq. (31) to obtain

33 $$\begin{aligned} g_2 = \frac{N/2 - g_1 \left[ F\left( r_{1} + L/2\right) - F\left( r_{1} - L/2\right) - F\left( 2r_{1}\right) + F\left( 0\right) \right] }{F\left( 2r_{1}\right) - F\left( 0\right) }. \end{aligned}$$

The bridge equilibrium may be characterized based on the behavior of this function for $g_2$. In particular, we will focus on the range of $g_2$ for which the bridge equilibrium is valid, as well as the number of bridge equilibria. The number of bridge equilibria corresponds to the number of solutions $r_{1}$ for a given $g_2$ in Eq. (33), i.e., the number of fixed points $r_1$ for a given $g_2$.

**Fig. 6:**
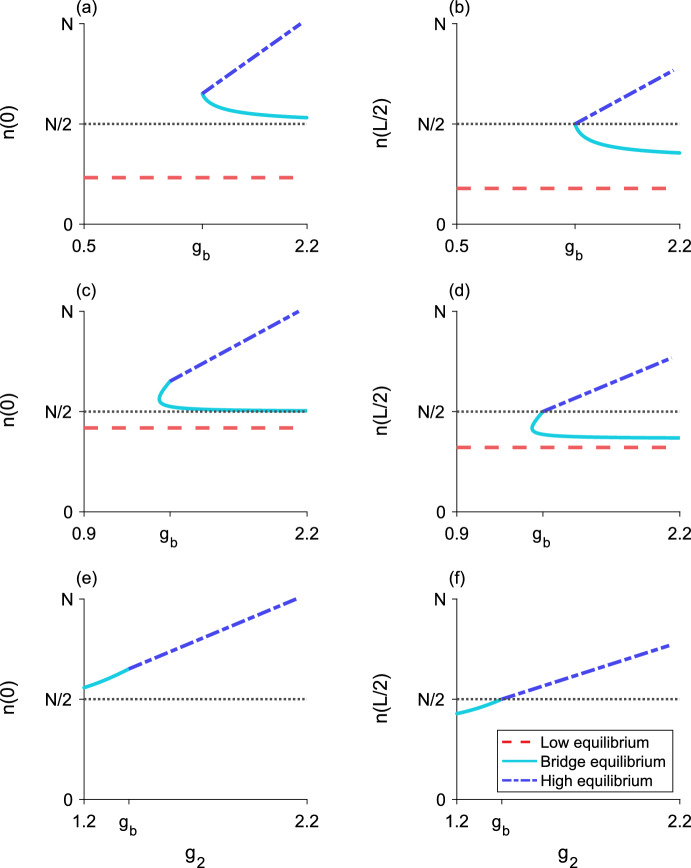
Bifurcation diagram as $g_2$ varies for three different values of $g_1$. The left column (**a, c, e**) plots the maximum population-density ${n\left( 0\right) }$ and the right column (**b, d, f**) plots the minimum population-density ${n\left( L/2\right) }$. The horizontal dotted line is the density-threshold value *N*/2. (**a, b**), ${g_1 = 0.5}$ and ${g_1 < g_{s}}$; there is a single unstable bridge-equilibrium valid for ${g_b< g_2 < g_c}$. (**c, d**), ${g_1 = 0.9}$ and ${g_{s}< g_1 < g_a}$; the upper branch of bridge equilibria are stable and the lower branch of bridge equilibria are unstable. (**e, f**), ${g_1 = 1.2}$ and ${g_a< g_1 < g_2}$; there is a single stable bridge-equilibrium valid for ${g_1< g_2 < g_b}$. Both the low and high equilibria are stable. Parameter values are ${L = 0.25}$, ${N = 1}$, ${\alpha = 5}$, ${g_{s} \approx 0.6}$, ${g_a \approx 1.1}$, ${g_b \approx 1.4}$, ${g_c \approx 2.2}$

To classify the range of valid $g_2$ values for the bridge equilibrium, we first observe that the bridge equilibrium meets the low equilibrium at ${r_{1} = 0}$. Using Eq. (33), we see that $g_2$ will then go to ${\pm \infty }$ depending on the value of $g_1$. In particular, for ${g_1 < g_a}$, ${g_2 \rightarrow \infty }$ while, for ${g_1 > g_a}$, ${g_2 \rightarrow -\infty }$. Next, we observe that the bridge equilibrium meets the high equilibrium at ${r_{1} = L/2}$ and ${g_2 = g_b}$ (see Eq. (25) and Fig. 4). These two observations make it clear that Eq. (33) may have one or two branches (see Fig. 6). As a result, there may be either one or two bridge-equilibria. Whether Eq. (33) is single- or multi-valued also depends on the relative size of $g_1$. Combined with the behavior of $g_2$ in Eq. (33) as ${r_{1} \rightarrow 0}$, there are three distinct parameter regimes with differing behavior of the bridge equilibria, summarized in Table 1.

For small $g_1$, the function for $g_2$ is single-valued, with ${g_2 \rightarrow \infty }$ as mentioned above (see Fig. 6a, b). Thus, there is a single bridge-equilibrium that is valid for ${g_2 > g_b}$.

The function for $g_2$ becomes multivalued as $g_1$ increases through the limit

34 $$\begin{aligned} g_1 = \frac{N k\left( L\right) }{\left[ F\left( L\right) - F\left( 0\right) \right] \left[ k\left( L\right) + k\left( 0\right) \right] } = g_{s}. \end{aligned}$$

As this is a switching point of the bridge equilibrium, rather than a point where two equilibria meet, we use the subscript *s* for ‘switch.’ We still have ${g_2 \rightarrow \infty }$, but there are two solutions $r_{1}$ for some ${g_2 < g_b}$ and a single solution for ${g_2 > g_b}$ (see Fig. 6c, d). Therefore we have two bridge-equilibria with distinct spatial thresholds for some ${g_2 < g_b}$; for ${g_2 > g_b}$ only one bridge equilibrium remains. For both prior cases in which ${g_2 \rightarrow \infty }$, to guarantee that ${n\left( 0\right) < N}$ the true upper limit in $g_2$ is $g_c$.

As $g_1$ increases further through $g_a$, the function becomes single-valued, but now ${g_2 \rightarrow -\infty }$ (see Fig. 6e, f). In practice, the lower bound on $g_2$ is $g_1$. There is once again a single bridge-equilibrium valid for ${g_1< g_2 < g_b}$.

These parameter regimes for the bridge equilibrium are illustrated in Fig. 4. These limits also encompass the restrictions that ${n\left( L/2\right)< N/2< n\left( 0\right) < N}$. In Fig. 4, note that for ${g_s< g_1 < g_a}$, increasing $g_2$ through the lower boundary of validity corresponds to a fold bifurcation leading to the emergence of two bridge-equilibria. The three behaviors of the bridge equilibrium are further demonstrated in the bifurcation diagrams of Fig. 6, showing both the maximum and minimum densities of the equilibria as $g_2$ varies for different $g_1$ values.

**Table 1:** Number of bridge equilibria in different parameter regions. If there is no entry in a particular row or column, there is no valid solution to Eq. (33) and therefore no bridge equilibrium in that region

	$g_1< g_2 < g_b$	$g_b< g_2 < g_c$
$0< g_1 < g_{s}$		One bridge-equilibrium
$g_{s}< g_1 < g_a$	Two bridge-equilibria for some $g_2<g_b^{\textrm{a}}$	One bridge-equilibrium
$g_a< g_1 < g_2$	One bridge-equilibrium	

$^{\textrm{a}}$ We have no analytical expression for the lower bound in $g_2$ when $g_2$ has two branches. There is, however, a numerically calculable lower bound in $g_2$. See Fig. 4 for a graphical representation of this lower bound

To complete the characterization of the bridge equilibria, we would like to classify their stability. To accomplish this, we return to the spatial-threshold map given by Eq. (30). As noted earlier, the stability of the spatial-threshold fixed-point corresponds to the stability of the entire spatial equilibrium. Using the spatial-threshold map, we can classify stability as with any difference equation. We differentiate the map with respect to $r_{1, \, t}$. Denoting ${r'_1 = dr_{1, \, t+1}/dr_{1, \, t}}$, after evaluating the derivative at the fixed point $r_1$ we obtain

35 $$\begin{aligned} r'_1 = \frac{\left( g_1 - g_2\right) \left[ k\left( 2r_1\right) + k\left( 0\right) \right] }{g_1\left[ k\left( r_{1} + L/2\right) - k\left( r_{1} - L/2\right) \right] + \left( g_2 - g_1\right) \left[ k\left( 2r_1\right) - k\left( 0\right) \right] }. \end{aligned}$$

If the magnitude of the derivative is smaller than $+1$, the equilibrium distribution is stable, otherwise it is unstable.

In general, we find that when ${g_1 < g_{s}}$, the bridge equilibrium is unstable. This scenario is shown in the bifurcation diagrams in Fig. 6a and b, where there is a single branch of bridge equilibria for ${g_b< g_2 < g_c}$. In contrast, when ${g_1 > g_a}$ the bridge equilibrium is stable (Fig. 6e, f).

For ${g_{s}< g_1 < g_a}$, there is a fold bifurcation at some critical $g_2$ value, corresponding to the $r_1$ value where ${r_1' = 1}$ (Fig. 6c, d). In particular, the lower boundary for the bridge equilibrium when ${g_{s}< g_1 < g_a}$ shown in Fig. 4 demarcates this fold bifurcation. For $g_2$ below the boundary, there is no bridge equilibrium; for $g_2$ above this boundary and below $g_b$, there are two bridge-equilibria. The bridge equilibrium with the larger $r_1$ fixed point, corresponding to the upper branch in the bifurcation diagram (Fig. 6c, d), is stable. This upper branch terminates at ${g_2 = g_b}$, where it becomes a stable high-equilibrium. The bridge equilibrium with the smaller $r_1$ fixed point, or the lower branch in the bifurcation diagram (Fig. 6c, d), is unstable. This behavior is also evident in Fig. 5b where there are two bridge-equilibria. For details of these stability calculations, see Section A.

#### Two-step block-pulse IDE: conclusions

In general, we see in Fig. 4 that there are regions in parameter space for which we might have only a single equilibrium, whether low, high, or bridge, as well as regions where two or all three equilibria coexist. With two possible bridge-equilibria, there are four distinct equilibrium-distributions, though only three may ever coexist for a single set of parameters. Bifurcation diagrams for the equilibria with fixed $g_1$ and varying $g_2$ are presented in Fig. 6, showing how the equilibrium distributions meet at the density-threshold values.

The three $g_1$ values illustrate the different behaviors that the bridge equilibria may exhibit. For small $g_1$ (${g_1 < g_s}$), all three equilibria may coexist. In this scenario, there is something like a fold bifurcation as $g_2$ increases, leading to the simultaneous emergence of a high equilibrium and a bridge equilibrium (Fig. 6a, b).

For moderate $g_1$ (${g_s< g_1 < g_a}$), a fold bifurcation results in two bridge-equilibria appearing as $g_2$ increases. As $g_2$ increases through $g_b$, the upper bridge-equilibrium turns into the high equilibrium as the edges of the population distribution rise above the density threshold *N*/2 (Fig. 6c, d).

For larger $g_1$ (${g_a< g_1 < g_2}$), the low equilibrium does not exist. There is a single branch of equilibria, beginning as a bridge equilibrium and turning into the high equilibrium as $g_2$ increases (Fig. 6e, f).

The stability of the different equilibria in this model follows the typical pattern of alternating stability observed in difference equations. That is, stable equilibria never exist next to each other, nor do unstable equilibria. Both of the one-growth-level equilibria, where the growth function crosses the line of equality in Fig. 3a along a flat constant, are stable. Thus, the analytical stability results for the bridge equilibria from Sect. 4.2.3 can also be inferred from the structure of the equilibria shown in Fig. 6.

### Three-step block-pulse IDE

We perform the same analysis for the three-step model, searching for any patterns that emerge among the three block-pulse models. We will use similar terminology as in Sect. 4.2, with the understanding that any repeated notation now refers to the new parameters and points in the three-step model, and not the analogous points from the two-step model. The three-step block-pulse IDE has growth function

36 $$\begin{aligned} g\left( n_t\right) \approx {\left\{ \begin{array}{ll} g_1, &{} 0 \le n_t< N/3 \\ g_2, &{} N/3 \le n_t< 2N/3 \\ g_3, &{} 2N/3 \le n_t < N, \end{array}\right. } \end{aligned}$$

with ${g_1< g_2 < g_3}$. This growth function is illustrated in Fig. 3b.

There are two density thresholds, ${n_t\left( x\right) = N/3}$ and ${n_t\left( x\right) = 2N/3}$, with corresponding spatial-threshold points ${x = \pm r_{1, \, t}}$ and ${x = \pm r_{2, \, t}}$. Depending on the location and existence of these spatial thresholds, there may be six different forms of the analytic map for the three-step block-pulse IDE (and therefore six different forms of equilibrium distributions). Table 2 illustrates the six possible orientations of the spatial-threshold points. The population may have a single growth-level in the patch, for three distinct forms, may see two growth-levels in the patch, for two more forms, or may see all three growth-levels inside the patch, for one final form of the IDE.

When discussing the equilibrium distributions for these different forms of the IDE, in general, we refer to an equilibrium solution where the population sees *p* growth-levels inside the patch as a *p*-growth-level equilibrium, or a *p*-level equilibrium for succinctness. We have ${1 \le p \le m}$, with *m* the total number of steps in the block-pulse approximation. In the three-step model, ${m = 3}$, and so we have one-level, two-level, and three-level equilibria.

We will address the one-level, two-level, and three-level equilibria individually at first, before summarizing the overarching dynamics and patterns of the three-step model. As in the two-step model, there are a number of critical threshold-values for the growth levels $g_i$ that correspond to changes in equilibrium behavior. These thresholds are summarized in Table 3.

**Table 2:** Orientations of the spatial thresholds in the three-step block-pulse IDE, and their corresponding equilibrium type. The configurations are based on if each threshold exists and, if it does, whether it exists inside the patch or outside the patch. If there is no entry, that configuration of thresholds is not possible

	No $r_{2, \, t}$	$r_{2, \, t}$ Inside	$r_{2, \, t}$ Outside
No $r_{1, \, t}$	Low		
$r_ {1, \, t}$ Inside	Low bridge	Full bridge	
$r_{1, \, t}$ Outside	Middle	High bridge	High

#### One-level equilibria: low, middle, and high equilibria

For populations with a single growth-level $g_i$, ${i = 1, 2, 3}$, within the patch, the densities in the patch lie between ${\left( i-1\right) N/3< n_t < iN/3}$. The corresponding IDEs are

37 $$\begin{aligned} n_{t+1}\left( x\right)&= g_i \int _{x - L/2}^{x + L/2} k\left( z \right) dz \nonumber \\&= g_i \left[ F\left( x + L/2\right) - F\left( x - L/2\right) \right] . \end{aligned}$$

Each of these three maps generates a stable equilibrium. These one-level equilibria occur at

38 $$\begin{aligned} n\left( x\right) = g_i \left[ F\left( x + L/2\right) - F\left( x - L/2\right) \right] . \end{aligned}$$

For ${i = 1, 2, 3}$, we call these the low, middle, and high equilibria.

**Fig. 7:**
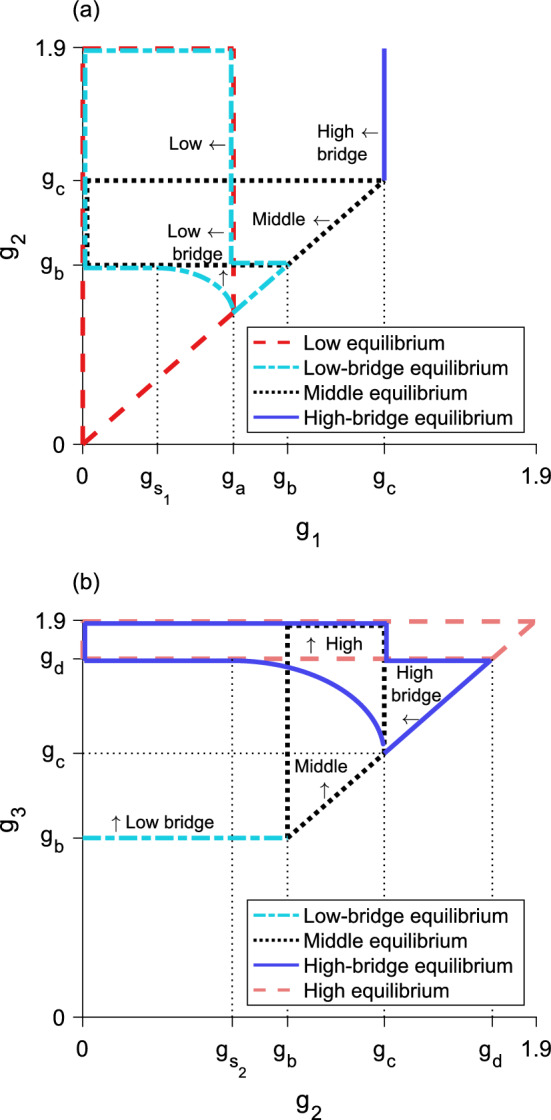
Regions of validity in **a** ${g_1 - g_2}$ space and **b** ${g_2 - g_3}$ space for five of the six equilibrium types of the three-step model. All equilibria are invalid for ${g_1 \ge g_2}$ and ${g_2 \ge g_3}$. **a**, region of validity is inside: the dashed region for the low equilibrium, the dotted region for the middle equilibrium, and the dashed-dotted region for the low-bridge equilibrium. The high-bridge equilibrium is valid to the left of the vertical solid line. For ${g_{s_1}< g_1 < g_a}$, as $g_2$ increases through the lower border of the region for the low-bridge equilibrium, a fold bifurcation occurs leading to two new low-bridge equilibria. **b**, region of validity is inside: the dotted region for the middle equilibrium, the dashed region for the high equilibrium, and the solid region for the high-bridge equilibrium. The low-bridge equilibrium is valid above the horizontal dashed-dotted line. For ${g_c< g_3 < g_d}$, as $g_2$ increases through the left boundary of the region for the high-bridge equilibrium, a fold bifurcation occurs leading to two new high-bridge equilibria. Parameter values are ${L = 0.3}$, ${N = 1}$, ${\alpha = 5}$, ${g_{s_1} \approx 0.3}$, ${g_{s_2} \approx 0.6}$, ${g_a \approx 0.6}$, ${g_b \approx 0.9}$, ${g_c \approx 1.3}$, ${g_d \approx 1.7}$, ${g_e \approx 1.9}$

The low equilibrium is valid so long as the maximum population-density does not exceed *N*/3, where the equilibrium transitions to a low-bridge equilibrium (Sect. 4.3.2). We thus require ${n\left( 0\right) < N/3}$, or ${g_1 < g_a}$, where $g_a$ is given in Table 3. Figure 7a shows the region in parameter space where this equilibrium is valid.

The middle equilibrium is valid if ${N/3< n\left( L/2\right)< n\left( 0\right) < 2N/3}$ (see Fig. 7), or when ${g_b< g_2 < g_c}$, with $g_b$ and $g_c$ as in Table 3. Violating the lower boundary pushes the population below *N*/3 at the boundaries and the equilibrium becomes a low-bridge equilibrium (Sect. 4.3.2). Violating the upper boundary moves the population above 2*N*/3 at the middle of the habitat and the equilibrium shifts to a high-bridge equilibrium (Sect. 4.3.2).

The high equilibrium is valid when ${n\left( L/2\right) > 2N/3}$ (see Fig. 7b), so that ${g_3 > g_d}$. Violating this condition leads to the equilibrium shifting to a high-bridge equilibrium (Sect. 4.3.2). We also require ${n\left( 0\right) < N}$, or ${g_3 < g_e}$. See Table 3 for the values of $g_d$ and $g_e$.

Though it may initially seem counterintuitive, the requirement that ${g_d< g_3 < g_e}$ can only be satisfied for small patch-lengths *L*. For larger *L*, ${g_e < g_d}$, and guaranteeing that the population is above 2*N*/3 everywhere also corresponds to the population being larger than the domain of population size *N*. Adjusting *N* does not affect this issue, as *N* is involved in both $g_d$ and $g_e$. This suggests that for larger patch-lengths, populations with large maximum densities are more likely to vary in density over the patch so that the equilibrium distributions have more than one growth-level inside the patch.

**Table 3:** Critical values of the growth levels $g_i$ for the three-step block-pulse IDE where equilibria meet or equilibrium behavior changes

$g_a = \frac{N/3}{F\left( L/2\right) - F\left( -L/2\right) }$	$g_{s_1} = \frac{\frac{2N}{3} k\left( L\right) }{\left[ F\left( L\right) - F\left( 0\right) \right] \left[ k\left( L\right) + k\left( 0\right) \right] }$
$g_b = \frac{N/3}{F\left( L\right) - F\left( 0\right) }$	$g_{s_2} = \frac{\frac{4N}{3}k\left( L\right) }{\left[ F\left( L\right) - F\left( 0\right) \right] \left[ k\left( L\right) + k\left( 0\right) \right] }$
$$g_c = \frac{2N/3}{F\left( L/2\right) - F\left( -L/2\right) }$$	
$$g_d = \frac{2N/3}{F\left( L\right) - F\left( 0\right) }$$	
$$g_e = \frac{N}{F\left( L/2\right) - F\left( -L/2\right) }$$	

#### Two-level equilibria: low-bridge and high-bridge equilibria

In the next two cases, the population satisfies ${\left( i-1\right) N/3< n_t\left( L/2\right) < iN/3}$ and ${iN/3< n_t\left( 0\right) < \left( i+1\right) N/3}$ for $i = 1, 2$. The population will see the two growth-levels $g_i$ and $g_{i+1}$ inside the patch. Using the general expression from Eq. (10), our population distributions in these scenarios will be

39 $$\begin{aligned} n_{t+1}\left( x\right)&= g_i \left[ F\left( x + L/2\right) - F\left( x -L/2 \right) \right] \nonumber \\&\quad + \left( g_{i+1} - g_i\right) \left[ F\left( x + r_{i, \, t}\right) - F\left( x - r_{i, \, t}\right) \right] . \end{aligned}$$

As with the bridge map in Sect. 4.2.3, we can define an implicit map to find the next spatial threshold $r_{i, \, t+1}$, and use this map to find the spatial-threshold fixed-points ${r_{i, \, t+1} = r_{i, \, t} = r_i}$. With the fixed points $r_i$, we may then use Eq. (39) to find the equilibrium distribution where ${n_{t+1} \left( x\right) = n_{t}\left( x\right) = n\left( x\right) }$. The two-level equilibrium that results is given by

40 $$\begin{aligned} n\left( x\right) = g_i \left[ F\left( x + L/2\right) - F\left( x -L/2 \right) \right] + \left( g_{i+1} - g_i\right) \left[ F\left( x + r_{i}\right) - F\left( x - r_{i}\right) \right] . \end{aligned}$$

We call this the low-bridge equilibrium for ${i = 1}$, and the high-bridge equilibrium for ${i=2}$.

For both the low-bridge and high-bridge equilibria, we seek to characterize the equilibria through the behavior of $g_2$. For the low-bridge equilibrium, utilizing Eq. (40) with ${i=1}$, fixing $g_1$ and using ${n\left( r_{1}\right) = N/3}$, we obtain an expression for $g_2$,

41 $$\begin{aligned} g_2 = \frac{N/3 - g_1\left[ F\left( r_{1} + L/2\right) - F\left( r_{1} - L/2\right) - F\left( 2r_{1}\right) + F\left( 0\right) \right] }{F\left( 2r_{1}\right) - F\left( 0\right) }, \end{aligned}$$

in terms of $r_{1}$.

**Fig. 8:**
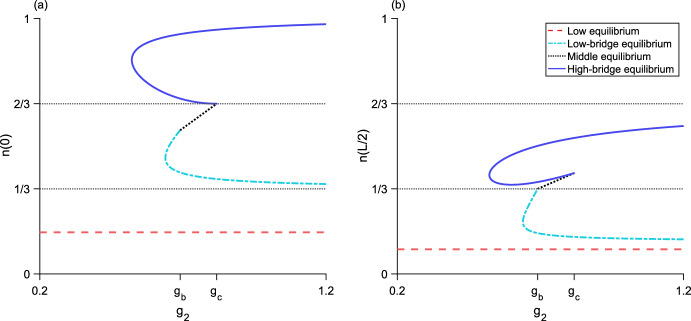
Bifurcation diagram as $g_2$ varies for the three-step model. **a**, the maximum population-density ${n\left( 0\right) }$ of the equilibrium distributions. **b**, the minimum population-density ${n\left( L/2\right) }$ of the equilibria. Neither the full-bridge nor high equilibria exist for this parameter set, while both the low-bridge and high-bridge equilibria have a fold bifurcation leading to two branches of equilibria. The low and middle equilibria, as well as the upper branches of both low-bridge and high-bridge equilibria, are stable. The lower branches of the low-bridge and high-bridge equilibria are unstable. Parameter values are ${L = 0.675}$, ${N = 1}$, ${\alpha = 5}$, ${g_1 = 0.2}$, ${g_3 = 1.2}$, ${g_{s_1} \approx 0.046}$, ${g_a \approx 0.41}$, ${g_b \approx 0.69}$, ${g_c \approx 0.82}$, ${g_d \approx 1.38}$

**Fig. 9:**
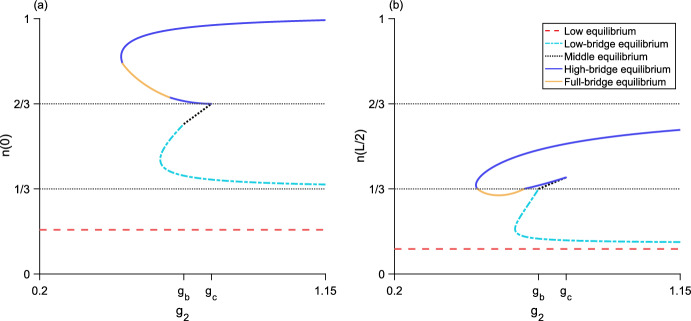
Bifurcation diagram as $g_2$ varies for the three-step model. **a**, the maximum population-density ${n\left( 0\right) }$ of the equilibrium distributions. **b**, the minimum population-density ${n\left( L/2\right) }$ of the equilibria. The high equilibrium does not exist for this parameter set, while both the low-bridge and high-bridge equilibria have a fold bifurcation leading to two branches of equilibria. Now, the full-bridge equilibrium is also valid. It exists as a single piece between two segments of the lower branch of the high-bridge equilibrium, where ${n\left( L/2\right) < N/3}$. The low and middle equilibria, as well as the upper branches of both low-bridge and high-bridge equilibria, are stable. The full-bridge equilibrium and lower branches of the low-bridge and high-bridge equilibria are unstable. Parameters are ${L = 0.8}$, ${N = 1}$, ${\alpha = 5}$, ${g_1 = 0.2}$, ${g_3 = 1.15}$, ${g_{s_1} \approx 0.024}$, ${g_a \approx 0.39}$, ${g_b \approx 0.68}$, ${g_c \approx 0.77}$, ${g_d \approx 1.36}$

**Fig. 10:**
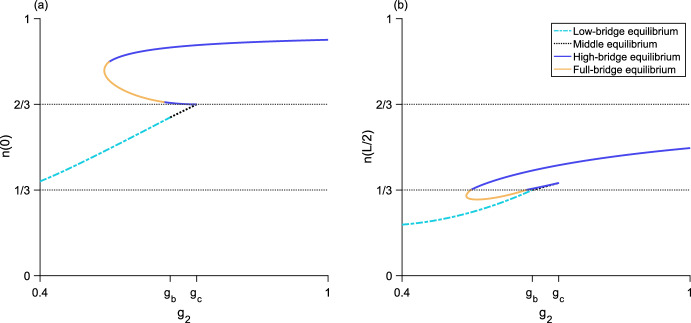
Bifurcation diagram as $g_2$ varies for the three-step model. **a**, the maximum population-density ${n\left( 0\right) }$ of the equilibrium distributions. **b**, the minimum population-density ${n\left( L/2\right) }$ of the equilibria. Neither the low or high equilibria exist for this parameter set. There is now only one low-bridge equilibrium. A fold bifurcation occurs in the full-bridge equilibrium, and there are two branches of high-bridge equilibria. The middle equilibrium and low-bridge equilibrium, as well as the upper branches of both high-bridge and full-bridge equilibria, are stable. The lower branches of the high-bridge and full-bridge equilibria are unstable. Parameters are ${L = 1}$, ${N = 1}$, ${\alpha = 5}$, ${g_1 = 0.4}$, ${g_3 = 1}$, ${g_a \approx 0.36}$, ${g_b \approx 0.67}$, ${g_c \approx 0.73}$, ${g_d \approx 1.34}$

**Fig. 11:**
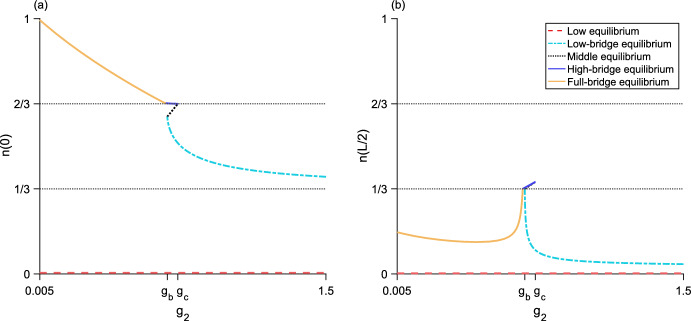
Bifurcation diagram as $g_2$ varies for the three-step model. **a**, the maximum population-density ${n\left( 0\right) }$ of the equilibrium distributions. **b**, the minimum population-density ${n\left( L/2\right) }$ of the equilibria. The high equilibrium does not exist for this parameter set. There is only one each of the low-bridge, high-bridge, and full-bridge equilibria. The low and middle equilibria are stable. The low-bridge, high-bridge, and full-bridge equilibria are unstable. Parameter values are ${L = 1}$, ${N = 1}$, ${\alpha = 5}$, ${g_1 = 0.005}$, ${g_3 = 1.5}$, ${g_{s_1} \approx 0.009}$, ${g_a \approx 0.36}$, ${g_b \approx 0.67}$, ${g_c \approx 0.73}$, ${g_d \approx 1.34}$

The behavior of this function for $g_2$ is nearly identical to the equivalent Eq. (33) for the bridge equilibrium in the two-step model. There are again three different parameter regimes leading to different behaviors of Eq. (41), and therefore different behaviors of the low-bridge equilibria. These parameter regimes for the low-bridge equilibria are summarized in Table 4 and displayed in parameter space in Fig. 7. Figures 8, 9, 10 and 11 show bifurcation diagrams of the maximum and minimum population-densities as $g_2$ varies, demonstrating the different behaviors of the low-bridge equilibria for different parameter sets. See Section C.1 for details of the behavior of Eq. (41) in the different parameter regimes.

The parameter limits in Table 4 encompass the restrictions ${n\left( L/2\right)< N/3 < n\left( 0\right) }$. At the point where the first inequality is violated, the low-bridge equilibrium meets the middle equilibrium. When the second inequality is violated, the low-bridge equilibrium meets the low equilibrium. There is a third restriction, ${n\left( 0\right) < 2N/3}$, to guarantee we have a low-bridge equilibrium. Violating this condition would result in the low-bridge equilibrium becoming a full-bridge equilibrium (Sect. 4.3.3). Given the other parameter restrictions for the low-bridge equilibrium to exist, this condition is always satisfied so that the low-bridge and full-bridge equilibria never meet.

**Table 4:** Number of low-bridge equilibria in different parameter regions. If there is no entry in a particular row or column, there is no valid solution to Eq. (41) and therefore no low-bridge equilibrium in that region

	$g_1< g_2 < g_b$	$g_b < g_2$
$0< g_1 < g_{s_1}^{\textrm{a}}$		One low-bridge equilibrium
$g_{s_1}< g_1 < g_a$	Two low-bridge equilibria for some $g_2<g_b^{\textrm{b}}$	One low-bridge equilibrium
$g_a< g_1 < g_b$	One low-bridge equilibrium	

${}^{\textrm{a}}$ See Table 3 for the value of $g_{s_1}$

${}^{\textrm{b}}$ There is a numerically calculable lower bound in $g_2$ for this region, shown in Fig. 7a, but no analytical expression for this lower bound

We now shift to examine what values of $g_2$ yield a valid high-bridge equilibrium, and the number of high-bridge equilibria. Utilizing Eq. (40) with ${i=2}$, fixing $g_3$ and using ${n\left( r_{2}\right) = 2N/3}$, we express $g_2$ as

42 $$\begin{aligned} g_2 = \frac{2N/3 - g_3\left[ F\left( 2r_{2}\right) - F\left( 0\right) \right] }{F\left( r_{2} + L/2\right) - F\left( r_{2} - L/2\right) - F\left( 2r_{2}\right) + F\left( 0\right) } \end{aligned}$$

in terms of $r_{2}$.

This function behaves similarly to the function for $g_2$ in the low-bridge equilibrium, though there are only two parameter regions that yield a valid high-bridge equilibrium. A summary of these parameter regimes is given in Table 5 and the regions are illustrated in Fig. 7. The bifurcation diagrams in Figs. 8, 9, 10 and 11 illustrate the behavior of the high-bridge equilibrium in these different parameter regions. The limits in Table 5 encompass the restrictions that ${n\left( L/2\right)< 2N/3< n\left( 0\right) < N}$. As in the other bridge equilibria, when the function for $g_2$ is multivalued, increasing $g_2$ through the lower boundary of validity for the high-bridge equilibrium corresponds to a fold bifurcation. See Section C.2 for a more detailed description of the behavior of Eq. (42) in each parameter region.

**Table 5:** High-bridge equilibria in different parameter regions. If there is no entry in a particular row or column, there is no valid solution to Eq. (42) and therefore no high-bridge equilibrium in that region

	$g_c< g_3 < g_d$	$g_d < g_3$
$0< g_2 < g_{s_2}^{\textrm{a}}$		One high-bridge equilibrium
$g_{s_2}< g_2 < g_c$	Two high-bridge equilibria for some $g_2^{\textrm{b}}$	One high-bridge equilibrium
$g_c< g_2 < g_d$	One high-bridge equilibrium	

${}^{\textrm{a}}$ See Table 3 for the value of $g_{s_2}$

${}^{\textrm{b}}$ A numerically calculable lower bound in $g_2$ exists for this region, shown in Fig. 7b, but there is no analytical expression for this lower bound

In addition to meeting the middle and high equilibria, the high-bridge equilibrium may also meet the full-bridge equilibrium if ${N/3 < n\left( L/2\right) }$ is violated. Earlier, we noted that the low-bridge and full-bridge equilibria do not meet. This is not true for the high-bridge equilibrium, and it is possible that ${n\left( L/2\right) = N/3}$ for ${r_{1} = L/2}$ and some $r_{2}$ value.

There are two main ways in which the high-bridge and full-bridge equilibria may interact, depending on whether there are one or two high-bridge equilibria. In both cases, it is possible that the full-bridge equilibrium is not valid so that the high-bridge and full-bridge equilibria do not meet. If the full-bridge equilibrium does exist, when there are two high-bridge equilibria there will be two $r_{2}$ values where ${n\left( L/2\right) = N/3}$ and the full-bridge equilibrium is spliced into the high-bridge equilibrium (Figs. 9 and 10). When there is a single high-bridge equilibrium, there may either be one (Fig. 11) or two $r_{2}$ values where ${n\left( L/2\right) = N/3}$. We will return to these interactions in Sect. 4.3.3.

To complete analysis of the low-bridge and high-bridge equilibria, we take the same approach to analyzing the stability of the equilibria as in Sect. 4.2.3, by considering the stability of the spatial-threshold fixed-points $r_{i}$, ${i = 1,2}$. The derivative of the spatial-threshold map, evaluated at $r_{i}$, is

43 $$\begin{aligned} r_i' = \frac{\left( g_i - g_{i+1}\right) \left[ k\left( 2r_i\right) + k\left( 0\right) \right] }{g_i\left[ k\left( r_i + L/2\right) - k\left( r_i - L/2\right) \right] + \left( g_{i+1} - g_i\right) \left[ k\left( 2r_i\right) - k\left( 0\right) \right] }, \end{aligned}$$

where ${r_i' = dr_{i, \, t+1}/dr_{i, \, t}}$. The equilibrium will be stable if ${|r_i' |< 1}$. Note this is the same general form of the derivative in Eq. (35) from Sect. 4.2.3.

When ${g_1 < g_{s_1}}$, the low-bridge equilibrium is unstable (Fig. 11). When ${g_1 > g_a}$, this equilibrium is stable (Fig. 10). For ${g_{s_1}< g_1 < g_a}$, there is a fold bifurcation for some $g_2$ value, corresponding to a critical $r_1$ value for which ${r_1' = 1}$. This bifurcation point in $g_2$ corresponds to the lower boundary for the low-bridge equilibrium in Fig. 7a when $g_1$ is within the given limits. As $g_2$ increases through this boundary, two low-bridge equilibria appear. The low-bridge equilibrium with the larger $r_1$ fixed point is stable, while the low-bridge equilibrium with the smaller $r_1$ fixed point is unstable. These correspond to the upper and lower branches of the low-bridge equilibria, as shown in Figs. 8 and 9.

For the high-bridge equilibrium, when ${g_c< g_3 < g_d}$, there is a fold bifurcation when ${r_2' = 1}$ (Figs. 8 and 9). This bifurcation point in $g_2$ corresponds to the left high-bridge boundary in Fig. 7b when $g_3$ is within the stated limits. As $g_2$ increases through the boundary, the fold bifurcation results in the appearance of two high-bridge equilibria. We note that for larger *L*, the fold bifurcation may occur in the full-bridge equilibrium instead, which also leads to two high-bridge equilibria (Fig. 10).

The high-bridge equilibrium with the larger $r_2$ fixed point, corresponding to the upper branch of the high-bridge equilibrium shown in Figs. 8, 9 and 10, is stable. The high-bridge equilibrium with the smaller $r_2$ fixed point, or the lower branch of the high-bridge equilibrium in Figs. 8, 9 and 10, is unstable. This lower branch terminates at ${g_2 = g_c}$ where it collides with the middle equilibrium.

For ${g_3 > g_d}$, the high-bridge equilibrium is unstable (Fig. 11). For details of the stability analysis for both two-level equilibria, see Section A.

#### Full-bridge equilibrium

In the sixth and final case, the population is below *N*/3 at the edges of the patch, above 2*N*/3 at the center of the patch, and between *N*/3 and 2*N*/3 in some interval between the edges and middle of the patch. The population sees all three growth-levels $g_1$, $g_2$, and $g_3$. The population distribution is

44 $$\begin{aligned} n_{t+1}\left( x\right)&= g_1 \left[ F\left( x + L/2\right) - F\left( x - L/2\right) \right] \nonumber \\&\quad + \left( g_2 - g_1\right) \left[ F\left( x + r_{1, \, t}\right) - F\left( x - r_{1, \, t}\right) \right] \nonumber \\&\quad + \left( g_3 - g_2\right) \left[ F\left( x + r_{2, \, t}\right) - F\left( x - r_{2, \, t}\right) \right] . \end{aligned}$$

We may use Eq. (44) to construct a two-dimensional map for the spatial thresholds $r_{1, \, t}$ and $r_{2, \, t}$. We can use the map to find the spatial-threshold fixed-points where both ${r_{1, \, t+1} = r_{1, \, t} = r_1}$ and ${r_{2, \, t+1} = r_{2, \, t} = r_2}$ are satisfied, and then use these fixed points to find the full equilibrium-distribution. This is the full-bridge equilibrium, given by

45 $$\begin{aligned} n\left( x\right)&= g_1 \left[ F\left( x + L/2\right) - F\left( x - L/2\right) \right] \nonumber \\&\quad + \left( g_2 - g_1\right) \left[ F\left( x + r_{1}\right) - F\left( x - r_{1}\right) \right] \nonumber \\&\quad + \left( g_3 - g_2\right) \left[ F\left( x + r_{2}\right) - F\left( x - r_{2}\right) \right] , \end{aligned}$$

which is valid for ${n\left( L/2\right)< N/3< 2N/3 < n\left( 0\right) }$.

As the full-bridge equilibrium depends on three growth-levels and two spatial thresholds, analysis becomes more challenging. It is more intuitive to understand the full-bridge equilibrium based on how it interacts with the high-bridge equilibrium. The full-bridge and high-bridge equilibria meet when ${n\left( r_{1} = L/2\right) = N/3}$, so that the high-bridge equilibrium transitions to the full-bridge equilibrium when

46 $$\begin{aligned} g_2\left[ F\left( L\right) - F\left( 0\right) \right] + \left( g_3 - g_2\right) \left[ F\left( L/2 + r_{2}\right) - F\left( L/2 - r_{2}\right) \right] = N/3, \end{aligned}$$

or, equivalently, when

47 $$\begin{aligned} g_2 = \frac{N/3 - g_3\left[ F\left( L/2 + r_{2}\right) - F\left( L/2 - r_{2}\right) \right] }{F\left( L\right) - F\left( 0\right) - F\left( L/2 + r_{2}\right) + F\left( L/2 - r_{2}\right) }. \end{aligned}$$

Equation (47), which only depends on one spatial threshold $r_{2}$, allows us to determine general patterns of the full-bridge equilibrium by observing the possible $g_2$ values where the high-bridge and full-bridge equilibria meet. As ${r_{2} \rightarrow L/2}$, ${g_2 \rightarrow -\infty }$. As ${r_{2} \rightarrow 0}$, ${g_2 \rightarrow g_b}$. Thus, we may infer that the full-bridge equilibrium is valid for at least some values ${g_2 < g_b}$.

**Fig. 12:**
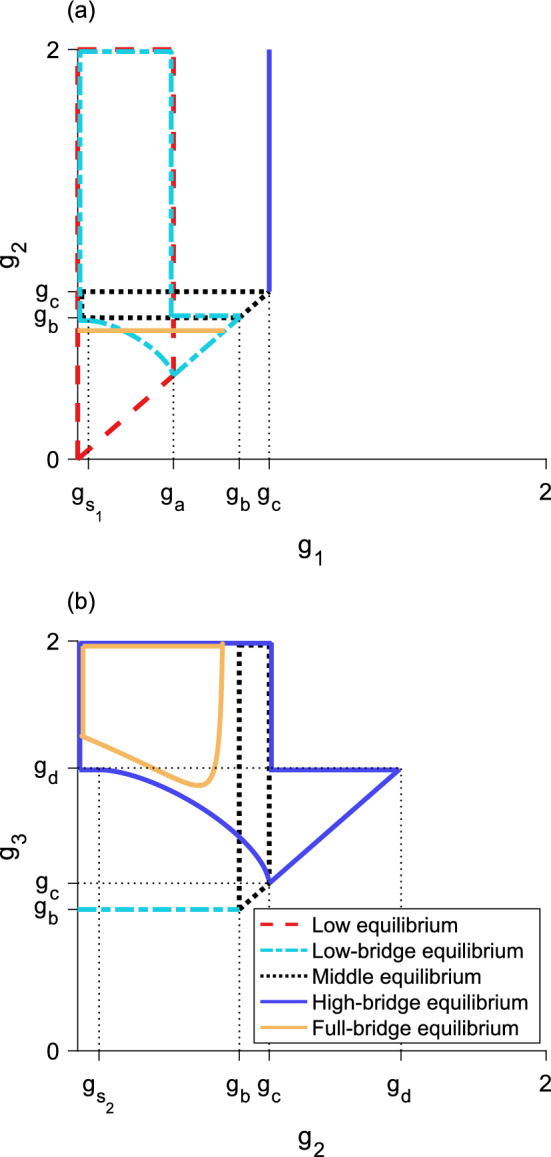
Regions of validity in **a** ${g_1 - g_2}$ space and **b** ${g_2 - g_3}$ space for the three-step model, with the region where the high-bridge and full-bridge equilibria meet. All equilibria are invalid for ${g_1 \ge g_2}$ and ${g_2 \ge g_3}$. A fold bifurcation occurs in the high-bridge equilibrium; for larger *L* the fold bifurcation may occur in the full-bridge equilibrium. **a**, the full-bridge equilibrium may be valid for $g_2$ below the nearly horizontal solid line. **b**, the high-bridge equilibrium is valid inside the outer solid region. The inner solid curve marks the boundary where the full-bridge equilibrium meets the high-bridge equilibrium. The full-bridge equilibrium exists inside the inner solid region. For fixed $g_3$, increasing $g_2$ through the left boundary of the region for the full-bridge equilibrium corresponds to the high-bridge transitioning to a full-bridge equilibrium. As $g_2$ increases through the right boundary of the region for the full-bridge equilibrium, there is a transition back into a high-bridge equilibrium. For this domain length, the high equilibrium is not valid. Parameter values are ${L = 0.675}$, ${N = 1}$, ${\alpha = 5}$, ${g_{s_1} \approx 0.05}$, ${g_{s_2} \approx 0.09}$, ${g_a \approx 0.4}$, ${g_b \approx 0.7}$, ${g_c \approx 0.8}$, ${g_d \approx 1.4}$

If there are two branches of high-bridge equilibria, the full-bridge equilibrium will meet a high-bridge equilibrium twice at two different $r_{2}$ values or not at all. These options are illustrated in Fig. 12b, when ${g_3 < g_d}$. In the latter case for smaller $g_3$ values, the full-bridge equilibrium is never valid (Fig. 8). In the former case for slightly larger $g_3$, the full-bridge equilibrium will exist in between two segments of the high-bridge equilibria. Figures 9 and 10 show two different ways in which this may occur. In Fig. 9, a fold bifurcation occurs in the high-bridge equilibrium, and the full-bridge equilibrium is spliced into the lower branch of the high-bridge equilibrium. In Fig. 10, there is a fold bifurcation in the full-bridge equilibrium instead, so that the upper full-bridge equilibrium meets the upper high-bridge equilibrium, and the lower full-bridge equilibrium meets the lower high-bridge equilibrium.

If there is a single high-bridge equilibrium for ${g_1< g_2 < g_c}$, the full-bridge equilibrium may meet the high-bridge equilibrium either twice or once for some ${g_2 < g_b}$. These possibilities are illustrated in Fig. 12b. That is, for ${g_3 > g_d}$, there are two options for crossing the inner solid boundary marking where the high-bridge and full-bridge equilibria meet (shown in Fig. 12b). For $g_3$ just larger than $g_d$, there is a high-bridge equilibrium that transitions into a full-bridge and then back again as $g_2$ increases. Thus, the full-bridge equilibrium exists between two segments of the high-bridge equilibrium. For relatively larger $g_3$, the equilibrium starts as a full-bridge equilibrium and turns into a high-bridge equilibrium (see Fig. 11). It may also be the case that Eq. (47) is never satisfied, so that the full-bridge equilibrium does not exist. This possibility exists for smaller *L*.

In general, we observe that if the full-bridge equilibrium exists and there are two branches of high-bridge equilibria, there may be one full-bridge equilibrium (Fig. 9) or two (Fig. 10). If there is only one high-bridge equilibrium, there will also only be one full-bridge equilibrium if it exists (Fig. 11).

We also use a stability analysis to cast light on the full-bridge equilibrium. With a two-dimensional mapping of the spatial thresholds, we employ the Jury conditions (Jury 1964), applied to the Jacobian of the map evaluated at the fixed point ${\left( r_1, \, r_2\right) }$. Three Jury conditions are needed for stability of a fixed point of this system, given by

48 $$\begin{aligned}{} & {} 1 - \tau + \Delta > 0, \end{aligned}$$

49 $$\begin{aligned}{} & {} 1 + \tau + \Delta > 0, \end{aligned}$$

50 $$\begin{aligned}{} & {} \Delta < 1, \end{aligned}$$

where $\tau$ and $\Delta$ are the trace and determinant of the Jacobian. The first condition ensures there are no eigenvalues greater than ${+1}$, the second that there are no eigenvalues less than ${-1}$, and the third that no complex eigenvalues are outside the unit circle. Violating any Jury condition leads to a local bifurcation and loss of stability.

Differentiating the two-dimensional spatial-threshold mapping with respect to $r_{1, \, t}$ and $r_{2, \, t}$ gives the Jacobian, from which we check the Jury conditions Eqs. (48)–(50). The map, Jacobian, and stability analysis may be found in Section B. We find that the eigenvalues of the Jacobian are always real. Thus, we do not need to consider the third Jury condition in Eq. (50), as this condition deals with complex eigenvalues. We also find that the second Jury condition in Eq. (49) is always satisfied. The only Jury condition that may be violated is the first. In particular, violation of this Jury condition leads to the fold bifurcation that we have already mentioned, where two distinct full-bridge equilibria emerge as $g_2$ increases (see Fig. 10).

In the case when ${g_3 > g_d}$, the full-bridge equilibrium is unstable. The only stable full-bridge equilibrium occurs if there is a fold bifurcation. In this case, the upper branch is stable and the lower branch is unstable. If a fold bifurcation occurs in the high-bridge equilibria and the full-bridge equilibrium exists between two segments of the lower high-bridge equilibrium, it is also unstable. These are largely heuristic results, and based primarily on continuity of stability for the high-bridge equilibria along with numerical experiments testing the first Jury condition for a wide range of parameter sets.

#### Three-step block-pulse IDE: conclusions

The three-step block-pulse IDE yields a remarkable complexity of behaviors. With six different types of equilibria and a variety of possible transitions between those equilibria, the dynamics of the system may be extremely varied, as seen in Figs. 8, 9, 10 and 11. There are potential fold bifurcations for three of the equilibria. That is, as $g_2$ (or any growth level) increases, there may be a fold bifurcation resulting in the sudden appearance of two new equilibria, one stable and one unstable. As the parameter increases further, there may also be another fold bifurcation in which an unstable equilibrium and a stable equilibrium collide in a sharp point and annihilate one another.

Stability of the equilibria again seems to follow a general trend. The one-level equilibria are always stable, and stability always alternates so that there are never two neighboring stable or unstable equilibria. In the case of a fold bifurcation, the upper equilibrium that emerges is stable, while the lower equilibrium is unstable. These patterns directly mirror the behavior of the two-step model.

## The *m*-step block-pulse IDE

Based on the trends observed in the one-, two-, and three-step block-pulse IDEs, we hypothesize that for an *m*-step block-pulse IDE the same stability patterns will continue. It is evident that the one-level equilibria, which have one growth-level $g_i$ inside the patch, are always stable. Stability of equilibria should also alternate between stable and unstable, and if a fold bifurcation occurs, the resulting upper branch of equilibria will be stable and the lower branch unstable. Based on the process outlined in previous sections for using the spatial-threshold fixed-points to find the full spatial equilibrium-distribution, we also construct a general expression for a *p*-level equilibrium,

51 $$\begin{aligned} n\left( x\right)&= g_q\left[ F\left( x + L/2\right) - F\left( x - L/2\right) \right] \nonumber \\&\quad + \sum _{i = q}^{q+p-2} \left( g_{i+1} - g_i\right) \left[ F\left( x + r_{i}\right) - F\left( x - r_{i}\right) \right] , \end{aligned}$$

for $1 \le p \le m$, where *q* is the index of the smallest growth-level, $g_q$, that the *p*-level equilibrium sees inside the patch.

The analytic expressions for the equilibria derived from Eq. (51) allow us to calculate the population densities of both stable and unstable equilibria for a block-pulse model. For each *m*-step block-pulse model, there are *m* one-level equilibria, ${m-1}$ two-level equilibria, and so on, for a total of ${m\left( m+1\right) /2}$ different equilibria. In general, there are ${m-p+1}$ different *p*-level equilibria for every ${1 \le p \le m}$.

For the *m* one-level equilibria, the population distributions are trivial to calculate using Eq. (51). For each of the ${m\left( m-1\right) /2}$ remaining *p*-level equilibria, ${2 \le p \le m}$, we may obtain the full population distribution by employing a multivariate root-finder to solve for the set of ${p-1}$ spatial-threshold fixed-points. Then, we substitute the fixed points into the relevant equilibrium distribution from Eq. (51) and evaluate over ${x \in \left[ -L/2, L/2\right] }$. Thus, we see the hybrid nature of this block-pulse method. Using the analytic expressions and patterns discovered in Sect. 4, we have generalized these patterns to the *m*-step case. To obtain the full equilibrium distributions of the block-pulse IDE, we use the analytic expressions for the equilibria as a basis and complete the process by using a numerical root-finder to solve for the small number of spatial-threshold fixed-points.

In our applications, to find the spatial-threshold fixed-points, we used MATLAB 2019a (MATLAB 2019) and the built-in function ‘fsolve,’ a function for solving a system of nonlinear equations by minimizing the sum of squares. We used this function with MATLAB’s trust-region dogleg algorithm, a robust and efficient iterative optimization-method for solving nonlinear systems of equations. (The open-source softwares Octave (Eaton et al. 2022) and R (R Core Team 2021) both have ‘fsolve,’ but we could not find the same trust-region dogleg algorithm in these packages.)

As there may be two branches of any given *p*-level equilibrium for ${p \ge 2}$, careful choice of initial guesses for the spatial thresholds is required to ensure that both solutions to the nonlinear system are reliably found. Choosing one initial guess to have the spatial thresholds close to *L*/2 and a second initial guess to have the spatial thresholds close to 0 is a good rule of thumb to ensure both branches, if they exist, of a *p*-level equilibrium are picked up. For example, in a three-level equilibrium, we might choose the first initial guess for the spatial thresholds to be ${\left( r_1, r_2\right) = \left( L/2 - \epsilon , \, L/2 - \epsilon \right) }$ and the second initial guess to be ${\left( r_1, r_2\right) = \left( \epsilon , \epsilon \right) }$ where $\epsilon$ is some small positive value.

## Applications

To illustrate the effectiveness of the block-pulse IDE, we applied three-, five-, and ten-step block-pulse models to an IDE with Beverton–Holt growth and an IDE with an Allee effect. For the original models, we calculated the maximum population-densities of both stable and unstable equilibria for varying growth-parameters using a phase-plane method, applicable for IDEs with the Laplace kernel (see, e.g., Kot and Schaffer 1986; Lutscher 2019). This method cannot, in general, be used for other kernels, which is why we chose the Laplace kernel for our examples.

For the block-pulse models, we calculated the peak densities of both stable and unstable equilibria using the process outlined in Sect. 5, evaluating Eq. (51) only at ${x = 0}$ after solving for and substituting the fixed points of a given equilibrium distribution into Eq. (51).

**Fig. 13:**
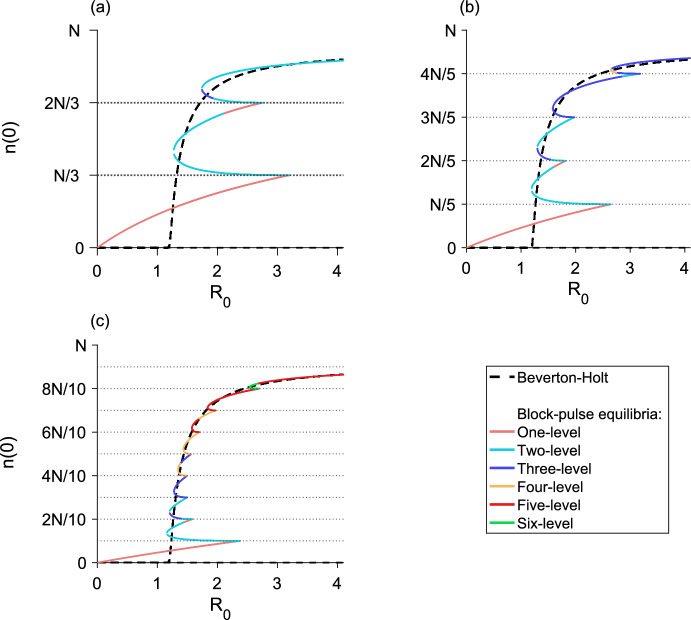
The maximum population-density as $R_0$ varies for the Beverton–Holt model (dashed) compared to a **a** three-step block-pulse model, **b** five-step block-pulse model, and **c** ten-step block-pulse model. All block-pulse models are shown with solid lines; *p*-level equilibria are distinguished from each other for each ${p = 1, 2,\ldots , m}$. The Beverton–Holt model has a stable trivial equilibrium until just past ${R_0 = 1}$; a transcritical bifurcation occurs as a positive stable branch of nontrivial equilibria emerges and the trivial branch of equilibria becomes unstable. In the block-pulse models, the lower branch of each fold bifurcation is unstable while the upper branches are stable, so that stability alternates. Horizontal dotted lines indicate the density thresholds for each block-pulse model. Parameter values are ${L = 1}$, ${N = 1}$, ${\alpha = 5}$, ${K = 1}$

**Fig. 14:**
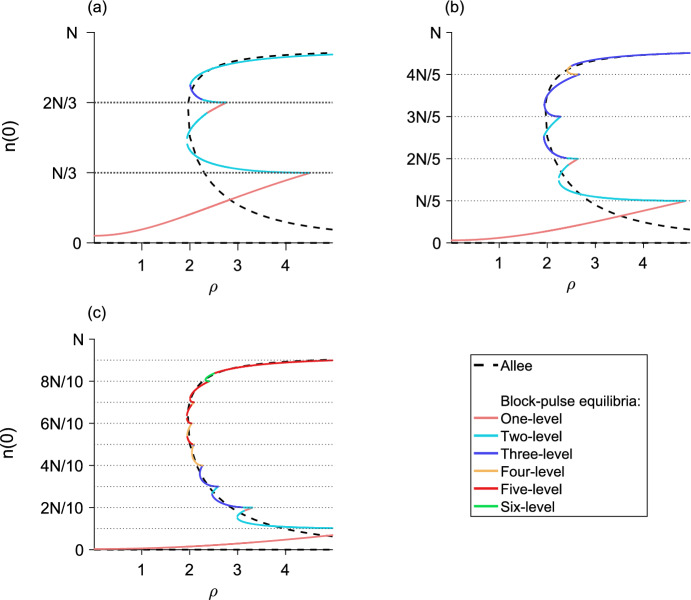
The maximum population-density as $\rho$ varies for an Allee-effect growth-model (dashed) compared to a **a** three-step block-pulse model, **b** five-step block-pulse model, and **c** ten-step block-pulse model. All block-pulse models are shown with solid lines; *p*-level equilibria are distinguished from each other for each ${p = 1, 2,\ldots , m}$. The lower branch of nontrivial Allee equilibria is unstable; the trivial and upper nontrivial branch of equilibria are stable. In the block-pulse models, for each fold bifurcation, the resulting lower branch is unstable and upper branch is stable, so that the lowest and highest branches of equilibria are stable, and stability alternates. Horizontal dotted lines indicate the density thresholds for each block-pulse model. Parameter values are ${L = 1}$, ${N = 1}$, ${\alpha = 5}$, ${K = 1}$

Bifurcation diagrams comparing the original models to block-pulse models as the growth parameter varies are shown for the Beverton–Holt model in Fig. 13 and for the Allee model in Fig. 14. Van Kirk and Lewis (1997) compute similar bifurcation diagrams using the average dispersal-success approximation. We therefore note that block-pulse IDEs are a complementary approach to methods like average dispersal-success or geometric symmetrization, and these methods may be used to answer similar ecological questions.

Sample code for computing the equilibrium distributions of a ten-step block-pulse model is available at https://doi.org/10.5281/zenodo.8153507. This code is intended as a starting point for the curious reader, and does not directly replicate any of the figures in this paper.

### Block-pulse IDE with Beverton–Holt growth

The Beverton–Holt model is a classical model of compensatory population growth given by

52 $$\begin{aligned} g\left( n_t\right) = \frac{R_0 n_t}{1 + \left[ \left( R_0 - 1\right) /K\right] n_t}, \end{aligned}$$

where *K* is the carrying capacity and $R_0$ is the net reproductive rate of the population. For the IDE with Beverton–Holt growth, there is a transcritical bifurcation in which a branch of nontrivial equilibria becomes positive, exchanging stability with the branch of trivial equilibria. A comparison of the maximum population-densities of the equilibria for the full growth-model and three-, five-, and ten-step block-pulse IDEs are shown in Fig. 13, where we distinguish between the different *p*-level equilibria of the block-pulse IDEs to illustrate where the types of equilibria occur and how they merge. For all three block-pulse models, a single fold bifurcation occurs between each set of density thresholds *iN*/*m* and ${(i+1)N/m}$, $i = 1, 2,\ldots , m-1$.

As the number of steps in the block-pulse model increases, we see increasing agreement between the equilibria for the two models. Numerically we observe that for each fold bifurcation in the block-pulse model, the upper branch of equilibria is stable and the lower branch is unstable. This is in agreement with our earlier conclusions regarding stability from Sect. 4. With an increasing number of steps, the block-pulse equilibria that trace the stable nontrivial equilibrium branch have longer and longer stable upper branches while the unstable portions get rapidly smaller. The lowest branch of equilibria also gets closer to zero, while the lowest fold bifurcation (where two equilibria collide and destroy each other) gets slowly closer to the point of bifurcation for the full Beverton–Holt model. For ten steps, the block-pulse model offers a decent approximation of the true equilibrium solutions, though it is not as accurate closer to the transcritical bifurcation in the full Beverton–Holt model.

For all three approximations, only a few equilibria have a large number of growth levels. In the three-step model, this is the three-level equilibrium; in the five-step model it is the four-level equilibrium; and in the ten-step model it is the six-level equilibrium. Indeed, for the ten-step model, even though there could be equilibria that have up to ten growth-levels, we see that nearly all of the equilibria have five or fewer growth-levels, and no equilibria have more than six growth-levels.

To assess numerical efficiency of the models, we also implemented three methods of solving the piecewise-defined IDE in Eq. (8) by iteration. We used a trapezoidal approximation to the integral, a convolution integral, and the CDF form of the model from Eq. (10) to eliminate integration entirely. In implementing the CDF form, we needed to update the spatial thresholds ${r_{i,t}}$ at each iterate. We did so by following the procedure for finding the spatial thresholds outlined in Sect. 5, that is, by using the root finder ‘fsolve.’ For comparison between these methods, we used a domain length of ${L = 1}$ and spatial grid spacing of ${2^{-10}}$. We simulated the model for 100 $R_0$ values with 100 different tent-map initial conditions each, running the three methods on a laptop with MATLAB 2019a (MATLAB 2019).

For a ten-step Beverton–Holt block-pulse model, the trapezoidal approximation with the built-in function ‘trapz’ took 5214 s to run. The convolution integral, using the built-in ‘conv’ function ran in 26 s. (The ‘trapz’ and ‘conv’ commands exist under the same names for Octave and R as well.) Using cumulative distribution functions to iterate the block-pulse model ran in 16 s, a more than 300-fold increase in speed over the simple trapezoidal approximation.

The full Beverton–Holt model, meanwhile, ran in 57 s using the built-in convolution integral. Thus, to obtain the stable equilibria of the model, the fastest method of implementation for a ten-step block-pulse model ran about 3.5 times faster than an extremely efficient method for simulating the original model, while still offering a good approximation to the original model.

The ten-step block-pulse equilibria calculated with analytic expressions ran in 303 s. This method is not directly comparable to the others as it calculates both stable and unstable equilibria.

### Block-pulse IDE with an Allee effect

We also applied three-, five-, and ten-step block-pulse models to an IDE with an Allee effect. The Allee growth-function was introduced in Sect. 2, with

53 $$\begin{aligned} g\left( n_t\right) = \frac{\left[ \left( 1 + \rho ^2\right) /K\right] n_t^2}{1 + \left( \rho /K\right) ^2 n_t^2}, \end{aligned}$$

where *K* is the carrying capacity and $\rho$ is the growth parameter. For the IDE with Allee effect, a fold bifurcation leads to the emergence of two branches of nontrivial equilibria, with a stable upper branch and unstable lower branch. There is always a stable branch of trivial equilibria. Figure 14 shows the maximum population-density of the equilibria as $\rho$ varies for the Allee IDE compared to the block-pulse IDEs, again illustrating where the different *p*-level equilibria occur and how they merge with one another. As in the Beverton–Holt model, a single fold bifurcation occurs between each set of density thresholds for each block-pulse model.

As the number of steps in the block-pulse model increases, we again see rapidly increasing agreement between the equilibria for the two different models. As in our analytics and the Beverton–Holt model, for each fold bifurcation that occurs in the block-pulse model as $\rho$ increases, the upper branch is stable and the lower branch is unstable. Furthermore, as the number of steps in the block-pulse model increases, the block-pulse equilibria that trace the stable nontrivial equilibrium branch have increasingly longer stable branches while the unstable portions get rapidly smaller. Similarly, the block-pulse equilibria that trace along the unstable branch of Allee equilibria start to have longer unstable branches while the stable sections become very small. The lowest, stable line of equilibria in the block-pulse model also gets closer to zero as the number of steps increase. For only ten steps, we have reasonably good agreement between the block-pulse model and the Allee model on both location and stability of the equilibria.

As in Sect. 6.1, for each block-pulse model, most equilibria have few growth-levels. For the ten-step model, we do find a six-level equilibrium, but that equilibrium only occurs over a very small range of $\rho$ values.

Using the same numerical methods as in the Beverton–Holt example, we also observed similar improvements in computational speeds for the Allee model. To obtain just the stable equilibria of the models, the CDF form of the ten-step block-pulse model ran a little over three times faster than the Allee model simulated with a convolution integral.

**Fig. 15:**
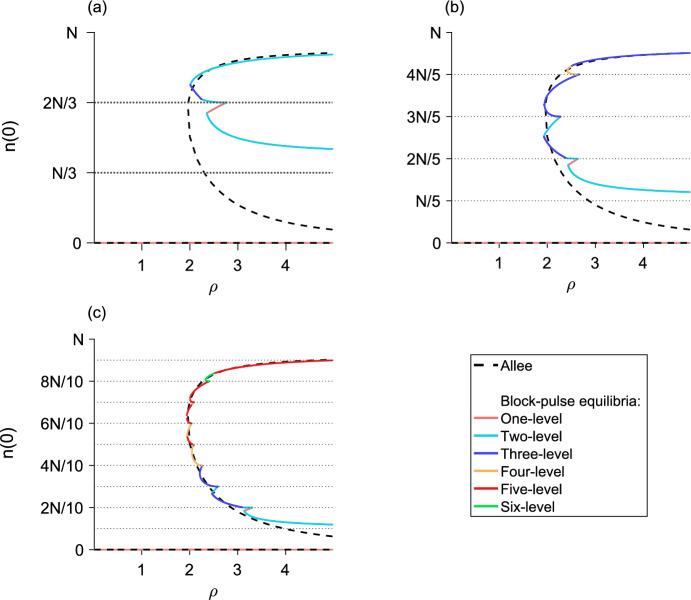
The maximum population-density as $\rho$ varies for an Allee-effect growth-model (dashed) compared to a **a** three-step block-pulse model, **b** five-step block-pulse model, and **c** ten-step block-pulse model. For the block-pulse models, we have set ${g_1 = 0}$ to allow for the possibility of extinction. All block-pulse models are shown with solid lines; *p*-level equilibria are distinguished from each other for each ${p = 1, 2,\ldots , m}$. The lower branch of nontrivial Allee equilibria is unstable; the trivial and upper nontrivial branch of equilibria are stable. In the block-pulse models, for each fold bifurcation, the resulting lower branch is unstable and upper branch is stable, so that the lowest and highest branches of equilibria are stable, and stability alternates. Horizontal dotted lines indicate the density thresholds for each block-pulse model. Parameter values are ${L = 1}$, ${N = 1}$, ${\alpha = 5}$, ${K = 1}$

As discussed earlier, the block-pulse models fail to account for the possibility of extinction, changing the bifurcation diagram so that the smallest equilibrium of the block-pulse model is never zero, unlike the true model. We briefly explored this issue by generating a bifurcation diagram for the Allee model as in Fig. 14, but with ${g_1 = 0}$. The results of this altered model are shown in Fig. 15.

Now, the desired extinction state is indeed present in the block-pulse model. The transitions between the equilibria are less smooth than in Fig. 14, though this improves as the number of terms increases. In general, the block-pulse equilibria still match the upper, stable branch of equilibria well, but there are much greater differences between the lower, unstable branch of equilibria and the block-pulse equilibria when there are only a few terms in the block-pulse model. This result is intuitive, as only the equilibria that depend on $g_1$ should be affected, and populations with smaller maximum densities are more likely to depend on $g_1$ than those with larger maximum densities. Furthermore, we see that agreement between the original model and the block-pulse model is reasonably good with more terms in the block-pulse series. So long as some sufficient number of terms are included in the block-pulse approximation, therefore, it seems reasonable to restrict ${g_1 = 0}$ instead of calculating $g_1$ according to Eq. (4).

**Fig. 16:**
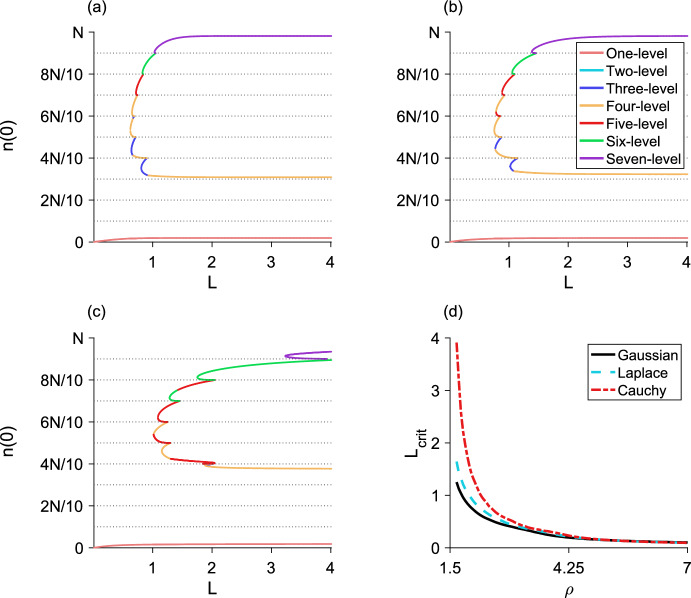
The maximum population-density as *L* varies for a ten-step block-pulse model with a **a** Gaussian, **b** Laplace, **c** Cauchy dispersal kernel, where ${\rho = 2.2}$. In **d**, the critical patch-size for population persistence is shown for the three dispersal kernels for varying growth-parameters $\rho$. To compare the three kernels, we used the same median absolute deviation (MAD) as a measure of dispersion. Parameters values are ${N = 1}$, ${K = 1}$, ${\alpha = 5}$, ${\eta = 0.05}$, ${\text {MAD} = 0.1386}$

We also applied a ten-step block-pulse model to the IDE with Allee effect where we varied the patch length *L* for a fixed growth-parameter $\rho$. The resulting equilibrium behavior is shown in Fig. 16 for three different dispersal kernels: the Gaussian (Fig. 16a), the Laplace (Fig. 16b), and the Cauchy (Fig. 16c) distributions. We set the mean or median of each kernel to zero, and we used the same median absolute deviation for all three kernels for consistency. As the unstable equilibria of the original Allee model cannot be computed for the Gaussian and Cauchy kernels, we do not show the equilibria of the original model in these examples.

In general, we observe that larger patch-lengths *L* tend to correspond to equilibria that have a larger number of growth levels. This matches our intuition from Sect. 4.3.1 that, for larger habitat-patches, population densities may be more likely to vary significantly over the patch.

While the bifurcation diagrams are reasonably similar for the Gaussian and Laplace kernels, the equilibrium behavior differs more significantly with the Cauchy kernel. In particular, the critical patch-length where a stable, nontrivial persistence-equilibrium emerges is larger for a model with a Cauchy dispersal kernel than the models with Gaussian or Laplace distributions. The maximum population-densities of the upper set of nontrivial equilibria are generally smaller, while the maximum population-densities of the lower set of nontrivial equilibria tend to be larger.

To compare the critical patch-size where a stable persistence-state first occurs more generally, we computed this critical patch-size for a variety of growth parameters $\rho$ for the three kernels (Fig. 16d). For the purposes of this problem, we defined the critical patch-length $L_\text {crit}$ as the minimum *L* value where a nontrivial equilibrium occurred. As our smallest estimated equilibrium is not quite zero, but is clearly approximating a trivial equilibrium, we add that in this case, ‘nontrivial’ means an equilibrium whose maximum density ${n\left( 0\right) > \eta }$, where $\eta$ is some small positive number. For an alternative approach to calculating the critical patch-size for the Laplace kernel, see Li and Otto (2022).

With this comparison, it appears that for a model with an Allee effect, the critical patch-length is somewhat sensitive to dispersal kernel for small growth-parameters $\rho$. For larger $\rho$, the difference in critical patch-length is negligible.

**Fig. 17:**
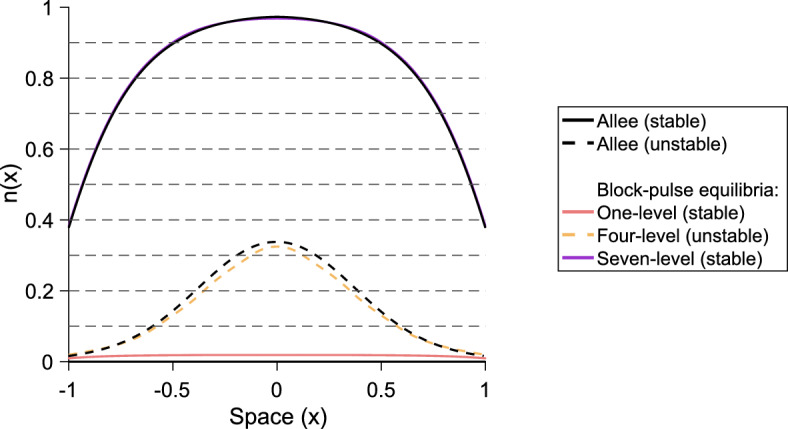
The full spatial equilibrium-distributions of the original Allee model compared to the block-pulse model, using the Laplace kernel, for ${\rho = 2.2}$ and ${L = 2}$. For the block-pulse model, these are the full equilibrium-distributions corresponding to the maximum population-densities at ${L = 2}$ shown in Fig. 16b. Stable equilibria are shown with solid curves; unstable equilibria are shown with dashed curves. Other parameter values are ${N = 1}$, ${K = 1}$, ${\alpha = 5}$

As a final illustration of the effectiveness of the block-pulse method, we compared the full spatial equilibrium-distributions of the original Allee model with those of the ten-step block-pulse model, using the Laplace kernel, for a given *L* and $\rho$ value (Fig. 17). In particular, we chose ${L = 2}$ and ${\rho = 2.2}$ for comparison to Fig. 16b, where we see the block-pulse model has one-level, four-level, and seven-level equilibria.

As can be seen in Fig. 17, the analytic equilibria of the block-pulse model very closely match those of the original Allee model. The largest stable equilibrium, in particular, is nearly indistinguishable between the two models. Thus, even for equilibrium population-distributions which vary significantly over their habitat patch, the block-pulse method offers close approximations to the full distributions. Furthermore, the block-pulse model reliably approximates distributions with different qualitative shapes, as shown in Fig. 17.

To highlight the simplicity of obtaining these equilibrium distributions, we explicitly provide the formula for the four-level unstable equilibrium shown in Fig. 17, as given analytically by Eq. (51). We also give the numerically-computed values of the three associated spatial-threshold fixed-points. The equilibrium is given by

54 $$\begin{aligned} n\left( x\right)&= g_1\left[ F\left( x + L/2\right) - F\left( x - L/2\right) \right] \nonumber \\&\quad + \sum _{i = 1}^{3} \left( g_{i+1} - g_i\right) \left[ F\left( x + r_{i}\right) - F\left( x - r_{i}\right) \right] , \end{aligned}$$

where we used the root-finding method discussed in Sect. 5 to solve for ${r_1 \approx 0.5758}$, ${r_2 \approx 0.3511}$, and ${r_3 \approx 0.1241}$. The $g_i$ values were computed using Eq. (4), with $g\left( n_t\right)$ as in Eq. (53) and the parameters taken from Fig. 17. In general, iterative methods for analyzing IDEs cannot pick up unstable equilibria at all. With the block-pulse method, we have explicit analytic expressions for both unstable and stable equilibria and gain insight into the spatial variation of the different equilibria, as demonstrated in Fig. 17.

## Discussion

In this work, we have presented a simple method for approximating the growth function in an IDE model to make analysis more tractable. The resulting block-pulse IDE has explicit analytic expressions for the iterates and equilibrium distributions of the model. For a small number of steps in the block-pulse series, we have fully described the possible equilibrium-solution behavior that emerges. These models have numerous different forms of equilibrium distributions, depending on how many density thresholds the population distributions pass through inside the spatial domain. These equilibria tend to merge with the neighboring equilibria, transitioning smoothly from one equilibrium type to the next as the population dips below a threshold at the edge of its habitat or rises above another threshold in the center of its patch.

Fold bifurcations, where two new branches of equilibria emerge as a growth parameter increases, are common features. As that parameter increases further, fold bifurcations in which two equilibria collide also occur, resulting in the abrupt loss of two equilibrium distributions. While this feature of fold bifurcations introduces excess solutions compared to the original model, we note that the range of parameter values where we have more solutions in the block-pulse model compared to the original model gets smaller as the number of steps increases, or as the original range fragments into smaller regions. Furthermore, given the patterns identified in the analytic expressions and stability, we may reasonably identify which solutions are extraneous, particularly as we observe how the different branches of solutions merge with each other and grow or become smaller as the number of terms in the model increases.

Stability of the equilibria seems to follow a predictable pattern. All one-level equilibria are stable. If two equilibria emerge from a fold bifurcation, the upper equilibrium is stable and the lower equilibrium is unstable. No two neighboring equilibrium-distributions have the same stability.

Block-pulse IDEs are generalizable to any increasing growth-function, given certain assumptions about the dispersal kernel and initial population-distributions. Thus, block-pulse IDEs are a highly useful method for analyzing many forms of population growth. Many methods exist for approximating equilibrium distributions and analyzing population persistence in IDEs, but these largely use approximations of or special forms of the dispersal kernel (Kot et al. 1996; Van Kirk and Lewis 1997; Lutscher and Lewis 2004; Kot and Phillips 2015; Reimer et al. 2016). In contrast, this is a novel formalization for a method that approximates the growth function in the IDE, though there are a few prior examples of piecewise-constant growth-functions in IDE models (Kot et al. 1996; Otto 2017; Nestor and Li 2022).

Block-pulse IDEs have a number of advantages inherent in their formulation. Chief among these is the ability to write down simple analytic expressions for the iterates of the model. Using these analytic expressions, we may obtain both unstable and stable equilibria of the block-pulse model. Furthermore, the equilibria of the block-pulse IDEs offer remarkably good approximations to the equilibria of the original models for only a modest number of terms in the block-pulse series. It is also straightforward to continually improve accuracy of the approximations by simply increasing the number of steps *m* in the model.

The analytic equilibrium expressions of the block-pulse IDE rely on the spatial-threshold fixed-points, which are found by solving a nonlinear system of implicit equations. To solve this system, we use a root-finding method. Other root-finding methods for finding the solutions of nonlinear operators, like IDEs, exist and have been in use for many years (Rall 1969; Krasnosel’skii et al. 1972; Hutson and Pym 1980; Werner 1981; Kantorovich and Akilov 1982; Precup 2002). These methods are, however, chiefly numerical and require solving for the population density at every location in the spatial domain.

In contrast, our analysis of a block-pulse IDE is a hybrid method. It is primarily analytical, but has a small numerical element. The numerical component requires solving for only a small number of spatial-threshold points, making this method computationally efficient compared to root-finding for operator equations. The block-pulse method also offers insight into the nature of the equilibrium distributions, showing the spatial variation of the equilibrium densities and how this variation changes with a parameter of the model. Furthermore, we may classify stability of the block-pulse equilibria, whereas operator root-finding methods do not easily allow for stability analyses.

We also note the effectiveness of using a block-pulse series to approximate IDEs with an Allee effect, a class of models that has long been challenging to investigate. In the invasion problem on the infinite domain, there are numerous prior works investigating Allee effects both analytically and numerically (Lui 1983; Kot et al. 1996; Veit and Lewis 1996; Wang et al. 2002; Fagan et al. 2005; Hurford et al. 2006; Goodsman and Lewis 2016; Sullivan et al. 2017; Nestor and Li 2022). On a finite domain, however, there is little research, aside from Van Kirk and Lewis (1997) and some numerical experiments (Etienne et al. 2002; Harsch et al. 2017), on the impact of an Allee effect in an IDE model. Block-pulse IDEs, therefore, offer a new method to explore Allee effects in IDEs.

We draw particular attention to the possibility of using block-pulse IDEs to estimate the critical patch-size *L* beyond which a stable persistence-state is possible. In general, the critical patch-size problem assumes there is no Allee effect (Lutscher 2019). As seen in Fig. 16, we may, however, estimate this critical patch-size by finding the smallest *L* such that a stable, nontrivial equilibrium exists.

Crucially, the critical patch-size appears to be somewhat sensitive to the choice of dispersal kernel at smaller growth-parameters, particularly if the kernel is heavy-tailed like the Cauchy kernel. This is in contrast to the result for models without an Allee effect, where critical patch-size does not depend significantly on the shape of the kernel for symmetric dispersal (Lockwood et al. 2002; Lutscher 2019). Thus, for kernels with heavier tails, the increased amount of long-distance dispersal may result in the population being pushed below the Allee threshold over a large enough portion of the habitat patch that extinction occurs.

As mentioned previously, block-pulse expansions may be applied to the dispersal kernel as well as, or instead of, to the growth function. For models with no Allee effect, the classic eigenvalue problem that emerges when studying the critical patch-size problem (Zhou and Kot 2013; Lutscher 2019) could potentially be analyzed by use of block-pulse expansions for the kernel as an alternative to current methods.

As we have noted already, block-pulse IDEs are a complementary approach to methods like average dispersal-success or geometric symmetrization, differing in which component of the IDE is approximated. Average dispersal-success, in particular, provides a leading-order approximation to the equilibrium population-size. If we were to combine the two approaches, using block-pulse functions for both the growth functions and to expand the kernel, we could feasibly obtain higher-order approximations to the equilibrium-solution distributions with a “full” block-pulse model where the entire integrand has been approximated by block-pulse functions.

Despite the advantages to block-pulse models, there are limitations of the current method. Any ecologically sound growth-function would have ${g\left( 0\right) = 0}$, so that a population cannot grow from nothing. The block-pulse framework, however, does not include this feature, so that extinction is never possible. Assuming the original growth-function does satisfy ${g\left( 0\right) = 0}$, we observe that as the number of steps *m* increase in the block-pulse model, we will have ${g_1 \rightarrow 0}$. As demonstrated in Fig. 15, an expedient solution is to set ${g_1 = 0}$ in the current model, rather than computing $g_1$ according to Eq. (4). Alternatively, the definition of the subintervals in the block-pulse series could be adjusted to restrict $g\left( 0\right) = 0$, though both options would take us away from the conventional definition of a block-pulse series.

The required assumptions about unimodality and symmetry in the dispersal kernels and population distributions restrict the types of problems this method can currently be used to analyze. We believe, however, that the results of this work demonstrate the practicality of the block-pulse method and that adapting the block-pulse framework to allow for asymmetric kernels and population distributions would be of immense benefit and a worthwhile pursuit. Indeed, initial numerical explorations into IDEs with a small amount of asymmetry in the kernel suggest that the block-pulse framework will be able to accommodate such problems quite well. Many current problems in spatial ecology, such as determining the speed of spread or finding conditions for population persistence in a moving habitat, have asymmetric population-distributions that our current model does not account for. If the restrictions on symmetry and unimodality can be loosened, we believe the block-pulse IDE may become even more useful.

Another key extension of the model is to use block-pulse IDEs in a two-species model that incorporates species interaction, with two-dimensional block-pulse approximations used for the growth functions. This method may be of particular use in the case that one or both species have an Allee effect in their growth.

In addition to the finite-habitat problems mentioned above, studying spreading rates on an infinite domain would also be valuable. An early, and successful, example of piecewise-constant growth-functions in an IDE was for a species with an Allee effect, examining symmetric spatial spread from a point release in an infinite habitat (Kot et al. 1996). In theory, we may be able to generalize an *m*-step block-pulse formulation for the same problem to gain deeper insight into spread rates for models with Allee effects, extending the finite habitat-patch framework.

The initial success of the work done by Mark Lewis on the infinite domain (Kot et al. 1996) and the results of this work suggest that block-pulse IDEs are a useful approximation method with potential applications to a variety of different problems, and that future work may explore what kinds of problems the block-pulse method is best suited towards. Block-pulse IDEs possess an appealing analytic tractability and an ability to find both stable and unstable equilibria. These models can shed light on spatial variation and stability of equilibrium distributions, and may be used to identify critical parameters for population persistence. Our work here formalizes this class of IDEs, providing a hybrid analytic-numerical method for examining the behavior of spatiotemporal population models.

## Data Availability

Data sharing not applicable to this article, as no datasets were generated or analyzed during the study.

## Appendix A: Two-level stability analysis

### A.1 Conditions for stability of two-level equilibria

We have already seen that in the two-step and three-step models, all of the one-level equilibria are stable. This leaves the two-level and three-level equilibria, where we analyze the stability of the spatial-threshold fixed-points $r_{i}$ as a proxy for stability of the full equilibrium-solution.

The bridge equilibrium from Sect. 4.2.3 and the low-bridge and high-bridge equilibria from Sect. 4.3.2 share many features. We first introduce a few key expressions that will be used in analyzing stability of all of these equilibria, that is, all the two-level equilibria.

First, we note that the derivatives $r_i'$, ${i = 1, 2}$, given in Eqs. (35) and (43) have the same general form. The magnitude of these derivatives governs stability of the two-level equilibria. Both numerator and denominator of Eqs. (35) and (43) are negative, so the derivative is positive for the two-level equilibria. Thus, if ${0< r_i' < 1}$, the fixed point is stable; if ${r_i' > 1}$, the fixed point is unstable.

For ${i = 1, 2}$, the derivative ${r_i' = 1}$ exactly if

A1 $$\begin{aligned} g_i\left[ k\left( r_i + L/2\right) - k\left( r_i - L/2\right) \right] + 2\left( g_{i+1} - g_i\right) k\left( 2r_i\right) = 0. \end{aligned}$$

If the left hand side of Eq. (A1) is positive, the equilibrium is unstable; if it is negative, the equilibrium is stable. We may rewrite this condition so that the derivative is equal to one if

A2 $$\begin{aligned} g_i = \frac{-2g_{i+1} k\left( 2r_i\right) }{k\left( r_i + L/2\right) - k\left( r_i - L/2\right) - 2k\left( 2r_i\right) } = g^*. \end{aligned}$$

for some $r_i$. If ${g_i < g^*}$, the two-level equilibrium is unstable. If ${g_i > g^*}$, the two-level equilibrium is stable.

The derivative of $g^*$ with respect to $r_i$ is negative everywhere, and so $g^*$ is a decreasing function of $r_i$. The maximum value of $g^*$ is at ${r_i = 0}$, so that

A3 $$\begin{aligned} g_{\text {max}}^* = \frac{-2g_{i+1} k\left( 0\right) }{k\left( L/2\right) - k\left( -L/2\right) - 2k\left( 0\right) } = g_{i+1}, \end{aligned}$$

where the second equality follows from the symmetry of the dispersal kernel, with ${k\left( L/2\right) = k\left( -L/2\right) }$. The minimum value of $g^*$ is at ${r_i = L/2}$, with

A4 $$\begin{aligned} g_{\text {min}}^* = \frac{2g_{i+1} k\left( L\right) }{k\left( L\right) + k\left( 0\right) }. \end{aligned}$$

### A.2 Bridge equilibrium

There are three distinct parameter regimes for the bridge equilibrium of the two-step model, summarized in Table 1. We approach stability of the bridge equilibria for each regime separately. The expressions from Eqs. (A1) to (A4) are applied with ${i = 1}$.

#### A.2.1 First region: small $g_1$

In the first region, ${g_1 < g_s}$ and ${g_2 > g_b}$. We note that

A5 $$\begin{aligned} g_2 > g_b = \frac{N/2}{F\left( L\right) - F\left( 0\right) }, \end{aligned}$$

so that

A6 $$\begin{aligned} g_{\text {min}}^* = \frac{2g_2 k\left( L\right) }{k\left( L\right) + k\left( 0\right) } > \frac{N k\left( L\right) }{\left[ F\left( L\right) - F\left( 0\right) \right] \left[ k\left( L\right) + k\left( 0\right) \right] } = g_s. \end{aligned}$$

Thus, ${g_1< g_s < g_{\text {min}}^*}$ for all ${r_1 \in \left( 0, \ L/2\right) }$. Therefore, the bridge equilibrium is unstable in this parameter regime.

#### A.2.2 Second region: moderate $g_1$

In the second region where ${g_s< g_1 < g_a}$ and ${g_1< g_2 < g_c}$, there are two $r_1$ equilibria, as seen earlier. We prove this explicitly by showing there is some $r_1$ value where ${r_1' = 1}$ and a fold bifurcation occurs in this parameter region.

First, note that at ${r_1 = 0}$, ${g_1 < g^*_{\text {max}} = g_2}$. Thus, the left-hand side of Eq. (A1) is clearly positive and the equilibrium is unstable. At ${r_1 = L/2}$, ${g_2 = g_b}$ and so ${g^*_\text {min} = g_s}$. As ${g_1 > g_s}$ in this parameter regime, we have ${g_1 > g^*_\text {min}}$. Thus, the left-hand side of Eq. (A1) is negative and the equilibrium is stable.

Thus, the left-hand side of Eq. (A1) goes from positive to negative as $r_1$ increases. Since $g^*$ is a decreasing function of $r_1$, there is a a single $r_1^*$ value where the left-hand side of Eq. (A1) is equal to zero, and therefore where ${r_1' = 1}$, leading to a fold bifurcation. For ${r_1 < r_1^*}$ the equilibria will be unstable; for ${r_1 > r_1^*}$ the equilibria will be stable. The smaller $r_1$ correspond to the lower branch of bridge equilibria and the larger $r_1$ to the upper branch of equilibria, so that the lower branch is unstable and the upper branch is stable.

#### A.2.3 Third region: large $g_1$

In the third parameter region, where ${g_a< g_1< g_2 < g_b}$, the equilibrium is not valid for all ${0< r_1 < L/2}$. Instead, there is some intermediate $r_1$ value, which we call $r_v$, where ${g_1 = g_2}$. For ${r_1 < r_v}$, ${g_2 < g_1}$ and the bridge equilibrium is invalid. For ${r_v< r_1 < L/2}$, the bridge equilibrium is valid.

We suspect the bridge equilibrium to be stable in this region. For this to be the case, we require that ${g_1 > g^*}$ from Eq. (A2) for all ${r_v< r_1 < L/2}$. As $g^*$ is a decreasing function of $r_1$, the maximum value $g^*$ can take on while the bridge equilibrium is still valid occurs at ${r_1 = r_v}$.

Thus, we need ${g_1 > g^*\left( r_1 = r_v\right) }$ for the bridge equilibrium to be stable. Checking this condition and using the fact that ${g_1 = g_2}$ at $r_v$, we find that if

A7 $$\begin{aligned} g^*\left( r_1 = r_v\right) = \frac{-2g_1 k\left( 2r_v\right) }{k\left( r_v + L/2\right) - k\left( r_v - L/2\right) - 2k\left( 2r_v\right) } < g_1, \end{aligned}$$

then

A8 $$\begin{aligned} g_1\left[ k\left( r_v + L/2\right) - k\left( r_v - L/2\right) - 2k\left( 2r_v\right) \right] < -2g_1 k\left( 2r_v\right) . \end{aligned}$$

Canceling like terms, this means that

A9 $$\begin{aligned} g_1\left[ k\left( r_v + L/2\right) - k\left( r_v - L/2\right) \right] < 0, \end{aligned}$$

which is clearly satisfied. Therefore, the bridge equilibrium is indeed stable for this parameter regime. This completes our stability analysis for the two-step model.

### A.3 Low-bridge equilibrium

There are three distinct parameter regimes for the low-bridge equilibrium of the three-step model, summarized in Table 4. The expressions from Eqs. (A1) to (A4) are applied with ${i = 1}$. The stability analysis is identical to the analysis for the bridge equilibrium, save for the coefficients of the threshold values $g_{s_1}$ and $g_b$, and so we do not repeat it here.

To summarize, in the first parameter regime where ${g_1 < g_{s_1}}$ and ${g_2 > g_b}$, the low-bridge equilibrium is unstable. In the second region where ${g_{s_1}< g_1 < g_a}$ and ${g_1 < g_2}$, a fold bifurcation occurs for some critical $r_1^*$ where ${r_1' = 1}$; for ${r_1 < r_1^*}$ the equilibria are unstable and for ${r_1 > r_1^*}$ the equilibria are stable. These are the lower and upper branches of low-bridge equilibria respectively. In the third parameter region where ${g_a< g_1< g_2 < g_b}$, the low-bridge equilibrium is stable.

### A.4 High-bridge equilibrium

For the high-bridge equilibrium of the three-step model, the expressions from Eqs. (A1) to (A4) are applied with ${i = 2}$. As before, we treat the stability for the two parameter regions from Table 5 separately.

#### A.4.1 First region: moderate $g_3$

In the first parameter regime where ${g_{s_2}< g_2 < g_d}$ and ${g_c< g_3 < g_d}$, there are two $r_2$ equilibria. We prove this explicitly by showing there is a fold bifurcation at some $r_2$ value such that ${r_2' = 1}$.

At ${r_2 = 0}$, ${g_2 < g^*_\text {max} = g_3}$. The left-hand side of Eq. (A1) is positive and the equilibrium is unstable. At ${r_2 = L/2}$, ${g_3 = g_d}$ and ${g_2 > g_{s_2} = g^*_\text {min}}$. The left-hand side of Eq. (A1) is negative and the equilibrium is stable.

Thus, a fold bifurcation occurs at some $r_2^*$ where the left-hand side of Eq. (A1) is equal to zero and therefore ${r_2' = 1}$. For ${r_2 < r_2^*}$, the equilibria are unstable and for ${r_2> r_2^*}$ the equilibria are stable. This means that the lower branch of high-bridge equilibria are unstable and the upper branch of equilibria are stable.

#### A.4.2 Second region: large $g_3$

In the second region, ${g_2 < g_c}$ and ${g_3 > g_d}$ and the high-bridge equilibrium is valid for only some ${0< r_2 < r_v}$. For ${r_2 > r_v}$ we have ${g_1 > g_2}$. We suspect the high-bridge equilibrium is unstable, so that we should have ${g_2 < g^*}$.

We drop all subscripts on *r* for the remainder of this section for notational convenience and to shorten the equations. Using the functional form for $g_2$ from Eq. (42), we thus want to show that

A10 $$\begin{aligned} \frac{2N/3 - g_3\left[ F\left( 2r\right) - F\left( 0\right) \right] }{F\left( r + L/2\right) - F\left( r - L/2\right) - F\left( 2r\right) + F\left( 0\right) } < \frac{-2g_3 k\left( 2r\right) }{k\left( r + L/2\right) - k\left( r- L/2\right) - 2k\left( 2r\right) }.\nonumber \\ \end{aligned}$$

Solving the above expression for $g_3$, for instability of the high-bridge equilibrium we require

A11 $$\begin{aligned} g_3 > \frac{\frac{2N}{3}\left[ k\left( r + L/2\right) - k\left( r - L/2\right) - 2k\left( 2r\right) \right] }{\left[ F\left( 2r\right) - F\left( 0\right) \right] \left[ k\left( r + L/2\right) - k\left( r - L/2\right) \right] - 2k\left( 2r\right) \left[ F\left( r + L/2\right) - F\left( r - L/2\right) \right] }.\nonumber \\ \end{aligned}$$

The above equation is an increasing function of *r*. This can be confirmed by taking the derivative of the right-hand side with respect to *r*, and simplifying to find that the derivative is always positive for ${0< r < L/2}$. The algebra to show this fact is straightforward but lengthy; we do not include it here.

Since the right-hand side of Eq. (A11) is an increasing function of *r*, the maximum value occurs for ${r = L/2}$. If $g_3$ is larger than this maximum value, Eq. (A11) will be satisfied for all ${0< r < L/2}$. Substituting ${r = L/2}$ into the right-hand side of Eq. (A11), our requirement becomes

A12 $$\begin{aligned} g_3 > \frac{\frac{2N}{3}\left[ k\left( L\right) + k\left( 0\right) \right] }{\left[ k\left( L\right) + k\left( 0\right) \right] \left[ F\left( L\right) - F\left( 0\right) \right] } = \frac{2N/3}{F\left( L\right) - F\left( 0\right) } = g_d. \end{aligned}$$

In this parameter regime, ${g_3 > g_d}$, and so the requirement is evidently satisfied. Thus, the high-bridge equilibrium is unstable in this parameter regime.

## Appendix B: Three-level stability analysis

The full-bridge equilibrium from the three-step model is a three-level equilibrium. With two spatial thresholds involved in the full-bridge equilibrium, stability is dependent on a two-dimensional mapping of the spatial thresholds, with the maps given by

B13 $$\begin{aligned} n_{t+1}\left( r_{1, \, t+1}\right)&= g_1\left[ F\left( r_{1, \, t+1} + L/2\right) - F\left( r_{1, \, t+1} - L/2\right) \right] \nonumber \\&\quad + \left( g_2 - g_1\right) \left[ F\left( r_{1, \, t+1} + r_{1, \, t}\right) - F\left( r_{1, \, t+1} - r_{1, \, t}\right) \right] \nonumber \\&\quad + \left( g_3 - g_2\right) \left[ F\left( r_{1, \, t+1} + r_{2, \, t}\right) - F\left( r_{1, \, t+1} - r_{2, \, t}\right) \right] = N/3, \end{aligned}$$

B14 $$\begin{aligned} n_{t+1}\left( r_{2, \, t+1}\right)&= g_1\left[ F\left( r_{2, \, t+1} + L/2\right) - F\left( r_{2,\,t+1} - L/2\right) \right] \nonumber \\&\quad + \left( g_2 - g_1\right) \left[ F\left( r_{2, \, t+1} + r_{1, \, t}\right) - F\left( r_{2, \, t+1} - r_{1, \, t}\right) \right] \nonumber \\&\quad + \left( g_3 - g_2\right) \left[ F\left( r_{2, \, t+1} + r_{2, \, t}\right) - F\left( r_{2, \, t+1} - r_{2, \, t}\right) \right] = 2N/3. \end{aligned}$$

To classify stability, we will compute the Jacobian of this two-dimensional map and analyze the Jury conditions (Jury 1964). The Jacobian is given by

B15 $$\begin{aligned} J = \begin{bmatrix} \frac{n_{11}}{d_1} &{} \frac{n_{12}}{d_1} \\ \frac{n_{21}}{d_2} &{} \frac{n_{22}}{d_2} \end{bmatrix}, \end{aligned}$$

where

B16 $$\begin{aligned} n_{11}= & {} \left( g_1 - g_2\right) \left[ k\left( 2r_1\right) + k\left( 0\right) \right] , \end{aligned}$$

B17 $$\begin{aligned} n_{12}= & {} \left( g_2 - g_3\right) \left[ k\left( r_1 + r_2\right) + k\left( r_1 - r_2\right) \right] , \end{aligned}$$

B18 $$\begin{aligned} n_{21}= & {} \left( g_1 - g_2\right) \left[ k\left( r_2 + r_1\right) + k\left( r_2 - r_1\right) \right] , \end{aligned}$$

B19 $$\begin{aligned} n_{22}= & {} \left( g_2 - g_3\right) \left[ k\left( 2r_2\right) + k\left( 0\right) \right] , \end{aligned}$$

B20 $$\begin{aligned} d_1= & {} g_1\left[ k\left( r_1 + L/2\right) - k\left( r_1 - L/2\right) \right] + \left( g_2 - g_1\right) \left[ k\left( 2r_1\right) - k\left( 0\right) \right] \nonumber \\{} & {} + \left( g_3 - g_2\right) \left[ k\left( r_1 + r_2\right) - k\left( r_1 - r_2\right) \right] , \end{aligned}$$

and

B21 $$\begin{aligned} d_2&= g_1\left[ k\left( r_2 + L/2\right) - k\left( r_2 - L/2\right) \right] + \left( g_2 - g_1\right) \left[ k\left( r_2 + r_1\right) - k\left( r_2 - r_1\right) \right] \nonumber \\&\quad + \left( g_3 - g_2\right) \left[ k\left( 2r_2\right) - k\left( 0\right) \right] . \end{aligned}$$

Notice that the numerators and denominators in the Jacobian are all negative, so that every term in the Jacobian is positive. The three Jury conditions involve the trace and determinant, $\tau$ and $\Delta$, of the Jacobian, where

B22 $$\begin{aligned} \tau = \frac{n_{11}}{d_1} + \frac{n_{22}}{d_2} \end{aligned}$$

and

B23 $$\begin{aligned} \Delta = \frac{n_{11} n_{22}}{d_1 d_2} - \frac{n_{12} n_{21}}{d_1 d_2}. \end{aligned}$$

First, we consider the Jury conditions that are not violated. The second Jury condition, ${1 + \tau + \Delta > 0}$, is always satisfied. Writing everything in a common denominator, we have

B24 $$\begin{aligned} 1 + \tau + \Delta = \frac{d_1 d_2 + n_{11} d_2 + n_{22} d_1 + n_{11} n_{22} - n_{12} n_{21}}{d_1 d_2}. \end{aligned}$$

As each $n_{ij}$ and $d_i$ is negative, their products are positive and all of the terms in the numerator of Eq. (B24) are positive except for the last, ${-n_{12} n_{21}}$. The denominator is positive. Thus, we need to show that the single negative term in the numerator is less than all the other terms.

To show the numerator of the second Jury condition is positive, we notice that ${-n_{12}n_{21}}$ has the positive factor ${\left( g_1 - g_2\right) \left( g_2 - g_3\right) }$. We look for other terms in the numerator with this factor, and show that the sum of all terms with the factor ${\left( g_1 - g_2\right) \left( g_2 - g_3\right) }$ is positive, which is sufficient to demonstrate the numerator as a whole is positive. Collecting these terms, for the numerator of the second Jury condition to be positive we need

B25 $$\begin{aligned}&\left[ k\left( 0\right) - k\left( 2r_1\right) \right] \left[ k\left( 0\right) - k\left( 2r_2\right) \right] + \left[ k\left( r_1 - r_2\right) - k\left( r_1 + r_2\right) \right] \left[ k\left( r_2 - r_1\right) - k\left( r_2 + r_1\right) \right] \nonumber \\&\quad + \left[ k\left( 2r_1\right) + k\left( 0\right) \right] \left[ k\left( 2r_2\right) + k\left( 0\right) \right] - \left[ k\left( r_1 + r_2\right) + k\left( r_1 - r_2\right) \right] \left[ k\left( r_2 + r_1\right) + k\left( r_2 - r_1\right) \right] \nonumber \\&\quad + \left[ k\left( 2r_1\right) + k\left( 0\right) \right] \left[ k\left( 0\right) - k\left( 2r_2\right) \right] + \left[ k\left( 2r_2\right) + k\left( 0\right) \right] \left[ k\left( 0\right) - k\left( 2r_1\right) \right] > 0. \end{aligned}$$

Since we assume the kernel is symmetric, ${k\left( r_1 + r_2\right) = k\left( r_2 + r_1\right) }$ and ${k\left( r_1 - r_2\right) }$ ${= k\left( r_2 - r_1\right) }$. Expanding and canceling like terms, the above condition becomes

B26 $$\begin{aligned} k^2\left( 0\right) > k\left( r_1 + r_2\right) k\left( r_1 - r_2\right) , \end{aligned}$$

which is evidently true. Thus, ${1 + \tau + \Delta > 0}$ and the second Jury condition is always satisfied.

The third Jury condition, ${|\Delta |< 1}$, is not applicable here, as it deals with complex eigenvalues. The eigenvalues of this system are real. We show this by computing the eigenvalues of the Jacobian using the characteristic equation ${\lambda ^2 - \tau \lambda + \Delta = 0}$, so that the eigenvalues are

B27 $$\begin{aligned} \lambda _{1,2} = \frac{1}{2}\left( \tau \pm \sqrt{\tau ^2 - 4\Delta }\right) . \end{aligned}$$

If the discriminant ${\tau ^2 - 4\Delta > 0}$, then the eigenvalues are real and we do not need to check the third Jury condition. We have

B28 $$\begin{aligned} \tau ^2 - 4\Delta&= \left( \frac{n_{11}}{d_1} + \frac{n_{22}}{d_2}\right) ^2 - 4\frac{n_{11} n_{22}}{d_1 d_2} + 4\frac{n_{12} n_{21}}{d_1 d_2} \nonumber \\&= \left( \frac{n_{11}}{d_1} - \frac{n_{22}}{d_2}\right) ^2 + 4\frac{n_{12} n_{21}}{d_1 d_2} > 0. \end{aligned}$$

Therefore, we have real eigenvalues and the third Jury condition will never be violated.

Finally, the first Jury condition, ${1 - \tau + \Delta > 0}$, may be violated. This is clearly demonstrated in Fig. 10, where we observe a fold bifurcation in the full-bridge equilibrium. In practice, this condition is largely useful in checking specific full-bridge equilibria for stability. Numerical experiments testing the first Jury condition for 80 parameter sets with full-bridge equilibria demonstrated that the upper branch of equilibria is stable, while the lower branch is unstable, just as in the low-bridge and high-bridge equilibria when a fold bifurcation occurred.

## Appendix C: Behavior of $g_2$ in the low-bridge and high-bridge equilibria

### C.1 Low-bridge equilibrium

The function for $g_2$ from Eq. (41) behaves nearly identically to that of the analogous function for the bridge equilibrium from Sect. 4.2.3. At ${r_{1} = 0}$, the low-bridge equilibrium meets the low equilibrium and ${g_2 \rightarrow \pm \infty }$. At ${r_{1} = L/2}$, the low-bridge equilibrium meets the middle equilibrium at ${g_2 = g_b}$. Equation (41) may also be multi- or single-valued in $r_{1}$.

For small $g_1$, the function for $g_2$ is single-valued with ${g_2 \rightarrow \infty }$ as ${r_{1} \rightarrow 0}$ (see Fig. 11). Therefore, we have a single low-bridge equilibrium valid for ${g_2 > g_b}$.

As $g_1$ increases through the limit ${g_1 = g_{s_1}}$ (see Table 3), Eq. (41) becomes multivalued (we again use a subscript *s* to denote that this is a switching point in the behavior of a single equilibrium; there is a second analogous switching-point for the high-bridge equilibrium). Now, ${g_2 \rightarrow \infty }$ and there are two $r_{1}$ solutions for some ${g_2 < g_b}$ (see Figs. 8 and 9). There will be two low-bridge equilibria for ${g_2 < g_b}$. One of these terminates at ${g_2 = g_b}$, leaving one remaining low-bridge equilibrium for ${g_2 > g_b}$.

As $g_1$ further increases through $g_a$, the function becomes single-valued again with ${g_2 \rightarrow -\infty }$; the actual lower limit for $g_2$ is $g_1$ (see Fig. 10). There is one low-bridge equilibrium for ${g_1< g_2 < g_b}$. For ${g_1 > g_b}$, the equilibrium becomes invalid as ${g_1 > g_2}$.

### C.2 High-bridge equilibrium

For the function for $g_2$ from Eq. (42), similar behavior as that of the function from Section C.1 occurs. At ${r_{2} = 0}$, the high-bridge and middle equilibria meet at ${g_2 = g_c}$. At ${r_{2} = L/2}$, the high-bridge equilibrium meets the high equilibrium and ${g_2 \rightarrow \pm \infty }$ depending on the value of $g_3$. The function may have one or two branches.

For ${g_3 < g_c}$, ${g_2 > g_3}$ and the high-bridge equilibrium is not valid. For ${g_c< g_3 < g_d}$, the function for $g_2$ has two branches, with ${g_2 \rightarrow \infty }$ (Figs. 8, 9 and 10). There are two high-bridge equilibria for some ${g_2 < g_c}$; for ${g_2 > g_c}$ only one equilibria remains. The lower bound in $g_2$ ranges from ${g_2 = g_{s_2}}$ up to ${g_2 = g_c}$. For ${g_3 > g_d}$, the function for $g_2$ is single-valued with ${g_2 \rightarrow -\infty }$; the lower limit of $g_2$ is effectively $g_1$ (Fig. 11). There is a single high-bridge equilibrium valid for ${g_1< g_2 < g_c}$.
